# Temperature-Dependent Infrared Engineering for Extreme Environments: All-Dielectric Thermal Photonic Metamaterials Stable at 1873 K in Air

**DOI:** 10.1007/s40820-025-02065-9

**Published:** 2026-01-21

**Authors:** Yang Liu, He Lin, Yunxia Zhou, Liming Yuan, Yanqin Wang, Xiaoliang Ma, Cheng Huang, Xiangang Luo

**Affiliations:** 1 State Key Laboratory of Optical Field Manipulation Science and Technology, Institute of Optics and Electronics, Chinese Academy of Sciences, Chengdu, 610209 People’s Republic of China; 2 College of Materials Sciences and Opto-Electronic Technology, University of Chinese Academy of Sciences, Beijing, 100049 People’s Republic of China; 3 School of Automation Engineering, University of Electronic Science and Technology of China, Chengdu, 611731 People’s Republic of China

**Keywords:** Extreme environment, Thermal photonic, Metamaterial, Machine learning, Thermal management

## Abstract

The development of infrared engineering technologies for extreme environments remains a formidable challenge due to the inherent trade-offs among optical performance, thermal stability, and mechanical integrity in thermal photonic metamaterials (TPMs). This work introduces a novel multi-objective design framework and demonstrates the design, fabrication, and validation of a TPM operating under extreme temperatures up to 1873 K. We have established a holistic design framework integrating temperature-dependent neural network and Pareto multi-objective optimization to co-optimize spectral response, component light-weighting, and structural efficiency. The framework achieves 100 times faster computation than genetic algorithms. The performance of the designed TPM was evaluated under various atmospheric models and detection distances. The TPM achieved a peak radiance suppression efficiency of 82% and a maximum attenuation of − 7.4 dB at 1200–1500 K. Experimentally, we fabricated an all-dielectric TPM using a refractory TiO_2_/BeO multilayer stack with only 5 layers and 2 μm total thickness. The optimized structure shows high reflectivity (0.62 at 3–5 μm; 0.48 at 8–14 μm) for radiative suppression and high emissivity (0.87 at 5–8 μm) for radiative cooling. The TPM withstands 1873 K for 12 h in air with less than 3% spectral drift, retaining excellent mechanical properties. On high-temperature components, it achieves 40–50% radiative suppression and 40–60 K (~ 10.1 kW m^−2^) radiative cooling at 1100 K, endures over 20 times thermal shock cycles (> 150 K s^−1^, 700–1500 K), and maintains stable performance over 5 cycles, with 78% visible and 98% microwave transmittance. This work establishes a new paradigm in the design and application of photonic materials for extreme environments.

**Supplementary Information:**

The online version contains supplementary material available at 10.1007/s40820-025-02065-9.

## Highlights

- Temperature-dependent infrared engineering integrated with Pareto multi-objective optimization to simultaneously co-optimize spectral response, component, and structural efficiency.
- All-dielectric thermal photonic metamaterials (TPMs, 5-layer & 2-μm) achieve 0.62/0.48 reflectivity (3–5/8–14 μm) for 82% radiative suppression (− 7.4 dB, 3–5 μm in atmosphere) and 0.87 emissivity (5–8 μm) for 10.1 kW m^−2^ radiative cooling.
- The integrated all-dielectric TPM delivers 40%–50% radiative suppression and 40–60 K cooling at 1100 K, withstands > 20 thermal shocks (> 150 K s^−1^), and maintains 78% visible & 98% microwave transmittance.

**Supplementary Information:**

The online version contains supplementary material available at 10.1007/s40820-025-02065-9.

## Graphical abstract

**Figure:**
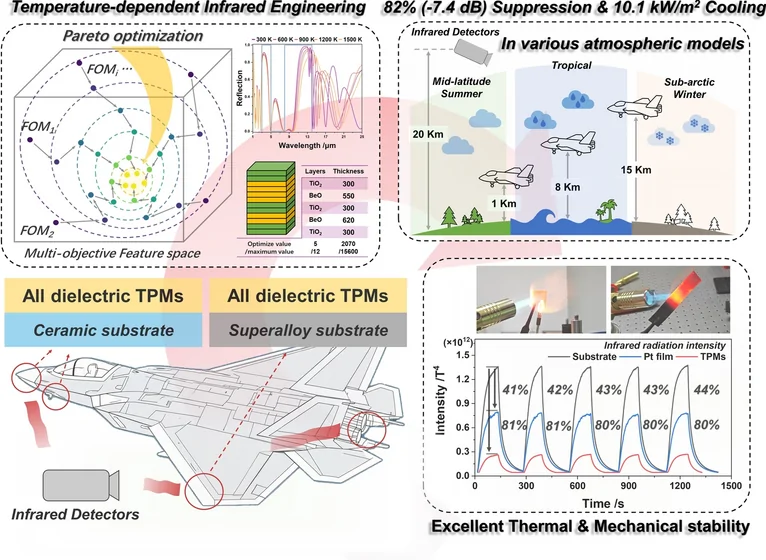

## Introduction

Artificially engineered thermal photonic metamaterials (TPMs) have brought revolutionary advancements to modern infrared radiation manipulation technologies through precise nanoscale light-matter control [1–8]. In the context of infrared camouflage, TPMs find applications ranging from the protection of critical facilities to enabling energy-efficient thermal management in aerospace systems [9–13]. The ultimate performance of TPMs depends on their spectral response within key atmospheric windows (3–5 and 8–14 μm) and heat-rejection capability in non-atmospheric windows (5–8 μm), thereby achieving a delicate balance between camouflage and radiative cooling [13]. However, the practical applicability of current thermal protection materials is severely constrained by the harsh realities of extreme environments. For aerospace vehicles, components such as wingtips and engine parts must endure harsh conditions including an oxidative atmosphere at temperatures surpassing 1500 K, severe fluid erosion, mechanical vibration, and extreme thermal shocks, leading to significant thermo-mechanical stress [14]. Although state-of-the-art TPMs demonstrate impressive spectral control at low to moderate temperatures [15–18], their performance deteriorates drastically beyond 1500 K.

Current strategies to mitigate these issues often involve protective coatings or inert atmospheres, which add complexity, weight, and potential failure points [19–26]. There is a critical, unmet need for a fundamental materials-by-design approach that co-optimizes optical function, thermo-mechanical stability, and chemical inertness from the ground up. This requires moving beyond traditional, sequential design, and adopting a holistic framework that treats high-temperature stability not as an afterthought but as a primary design objective [27–33]. For hypersonic aerospace vehicles, conventional infrared stealth fabrics used in ground equipment and heavy infrared stealth coatings are clearly inadequate. Infrared multilayered TPMs—capable of in situ integrated fabrication with component surfaces and suitable for large-area processing—represent the optimal solution. Unprotected Ag-/Ge-based dual-band infrared stealth TPMs, however, degrade at 523 K, likely due to high-temperature oxidation of Ag or Ge [13]. Incorporating aerogel thermal insulation allows ZnS-/Ge-based TPMs to operate up to 873 K, though their practical integration remains challenging for hypersonic vehicle applications [11]. The TPMs based on Sm^3+^/Ca^2+^ co-doped CeO_2_ achieve a markedly enhanced high-temperature infrared stealth capability by synergistically engineering both the oxygen vacancy concentration and the bandgap structure. This strategic design yields an exceptionally low infrared emissivity of 0.208 at 873 K [34]. Similarly, the use of SiO_2_ fiber paper supports TPM operation up to 1473 K, but the inherently porous and mechanically compliant structure hinders robust integration with hypersonic platforms [19]. Recently developed HfO_2_-/Mo-, Ge-/Mo-, and Si-/Mo-based TPMs have achieved effective infrared camouflage at 973 K [35–41]. Nevertheless, these systems face oxidation of Mo in atmospheric environments at higher temperatures. Additionally, due to the microwave-reflective nature of metallic Mo, achieving multispectral compatibility necessitates additional design strategies to ensure sufficient microwave transmission. Therefore, it is evident that current infrared stealth TPMs for extreme environments still face the following persistent challenges: (1) Poor high-temperature stability of metal/semiconductor materials such as Ge, ZnS, and Mo, which limits their applicability in infrared stealth under extreme environments; (2) The influence of temperature-induced photon–phonon–carrier coupling effects in these materials—current TPM designs do not account for temperature-dependent variations in phonon or charge carrier distributions, making it difficult to predict their high-temperature performance; (3) A significant lack of holistic design and experimental validation regarding reliability under extreme conditions, including thermal stress matching, fracture toughness, and interfacial adhesion strength of TPMs.

The structural and functional stability of TPMs under extreme conditions—encompassing high temperatures, high-speed gas flow erosion, and intense thermal shocks—is critically governed by two key geometric parameters: total thickness and number of layers. Excessive total thickness significantly compromises thermo-mechanical stability [42]. Under rapid thermal cycling or shock, a large thickness exacerbates the mismatch in the coefficient of thermal expansion (CTE) between the film stack and the substrate, generating substantial interfacial shear stress. This can lead to delamination, cracking, or even spallation failure. Furthermore, in high-speed airflow environments, thicker films are more susceptible to erosion and possess higher moment arms under aerodynamic shear forces, increasing the risk of mechanical peeling. From a functional perspective, although a larger optical path length can enhance interference effects, it also intensifies the negative impact of inherent material absorption and temperature-dependent refractive index drift, leading to spectral performance degradation at high temperatures. An excessive number of layers introduces multiple vulnerable interfaces, each being a potential site for interdiffusion, solid-state reactions, and defect formation [43]. At elevated temperatures, these interfaces become hotspots for atomic interdiffusion, which can disrupt the precisely engineered optical resonances, causing significant spectral drift (e.g., a decrease in reflectivity within 3–5 and 8–14 μm camouflage bands). Moreover, multilayer structures with numerous interfaces exhibit complex stress distribution and are highly prone to interfacial cracking under combined thermal and mechanical loads, ultimately leading to structural failure. Conversely, an over-pursuit of minimalism—too few layers or an insufficient total thickness—fails to provide the necessary optical phase modulation and Fabry–Perot resonance conditions. This results in poor spectral performance (e.g., insufficient reflectivity in the camouflage bands or inadequate emissivity in the radiative cooling band), rendering the TPM functionally ineffective from the outset.

Beyond the design of the metamaterial itself, the selection of a scalable and industrially viable fabrication technique is crucial for the practical deployment of TPMs on aerospace components. In this work, we employ magnetron sputtering for the deposition of the all-dielectric multilayer stack. Compared to other micro-/nano-fabrication techniques, such as laser processing or electron-beam lithography, magnetron sputtering offers distinct advantages for this application, including lower cost, higher throughput, excellent uniformity over large areas, and superior reproducibility. In fact, our prior work has already encompassed relevant efforts, including the fabrication of multilayer thin-film structured temperature sensors on a representative complex-shaped hot-section component—turbine rotor blades—as well as the development of similar sensor arrays on Hastelloy substrates compatible with large-scale roll-to-roll deposition processes [44, 45]. The inherent scalability of sputtering, combined with the robust all-dielectric material system presented here, provides a clear and practical pathway for transitioning our laboratory-scale TPMs to real-world, non-planar aerospace components, thereby addressing a key challenge in the field of thermal photonics for extreme environments.

Herein, we introduce a temperature-dependent infrared engineering strategy guided by Pareto multi-objective optimization. This computational framework simultaneously negotiates the often-contradictory constraints of optical performance, layer thickness, and structural simplicity to design all-dielectric metamaterials. The proposed framework achieves a computational speed 100 times faster than conventional genetic algorithms (9.1 vs. 970 ms). The fabricated TPMs are engineered to provide dual-band infrared stealth (high reflectivity of 0.62 and 0.48 in the 3–5 and 8–14 μm bands, respectively) coupled with efficient radiative cooling (high emissivity of 0.87 in the 5–8 μm band). The TPM achieved a peak radiance suppression efficiency of 82% and a maximum attenuation of − 7.4 dB at 1200–1500 K. We demonstrate that these materials withstand 1873 K in air for over 12 h with negligible (< 3%) spectral drift, maintaining structural integrity with over 80% retention of fracture toughness and interfacial adhesion. Furthermore, when deployed on aerospace-grade substrates, a thermally grown oxidation process was introduced to mitigate thermal stress mismatch at the metal–ceramic interface. The resulting TPMs exhibit significant radiative suppression (40%–50%) and passive cooling (40–60 K) at 1100 K, while also offering exceptional visible light (78%, 400–800 nm) and microwave (98%, X-band) transmittance for multispectral compatibility.

## Methods

### Computational Resources and Settings

The first-principles calculations presented in this study were performed on a cluster at the National Supercomputing Center, equipped with Hygon 7285H 32C CPUs operating at 2.50 GHz. Dataset preparation, neural network (NN) training, and the genetic algorithm combined with neural network (GA-NN) inverse design algorithm were executed on a personal computer (Dell G16 7630) featuring an Intel® Core™ i9-13900HX CPU (13th Gen, 2.20 GHz) and an NVIDIA GeForce RTX 4070 Laptop GPU. For further details regarding the first-principles calculations, ab initio molecular dynamics, dataset parameters, fully connected neural network architecture, and computational efficiency, refer to our previous research [46].

### Theoretical Model for Temperature-Dependent Infrared Spectra

To compute the full infrared spectral dielectric function (ε) at finite temperatures (300–1500 K), the Lorentz oscillator model was employed, expressed as Eq. (1):

1 $$\varepsilon \left(\omega \right)={\varepsilon }_{\infty }\prod_{j}\frac{{\omega }_{j,LO}^{2}-{\omega }^{2}+i{\gamma }_{j,\mathrm{LO}}\omega }{{\omega }_{j,TO}^{2}-{\omega }^{2}+i{\gamma }_{j,\mathrm{TO}}\omega }$$

where ${\varepsilon }_{\infty }$ represents the optical dielectric constant; j denotes infrared-active optical branches (longitudinal $\mathrm{LO}$ and transverse $\mathrm{TO}$ after Born effective charge and splitting corrections); ${\omega }_{j}$ is the phonon resonance frequency; ${\gamma }_{j}$ is the phonon damping factor for the jth infrared-active phonon mode, and $\varepsilon \left(\omega \right)$ is the sum of all infrared-active phonon resonances; and ${\gamma }_{j}$ is the sum of the third- and fourth-order phonon scattering rates (γ^3th^, γ^4th^). Notably, γ^3th^∝T and γ^4th^ ∝ ${T}^{2}$, with γ^4th^ becoming significant at high temperatures. To obtain the phonon frequencies and high-order phonon scattering rates across the 300–1500 K temperature range, the temperature-dependent effective potential (TDEP) method, based on anharmonic phonon self-energy calculations, was employed [47–49].

### Neural Network Architecture and Training Parameters

A dataset comprising 600,000 samples was utilized for neural network training. This dataset included 300,000 initial configurations of TPMs with layer thicknesses ranging from 100 to 1300 nm, supplemented by an additional 300,000 configurations where layer thicknesses were distributed across six specific intervals: 100–300, 300–500, 500–700, 700–900, 900–1100, and 1100–1300 nm (60,000 samples per interval). The sample temperatures were uniformly distributed across five levels: 300, 600, 900, 1200, and 1500 K. Spectral data covered the 1 to 25 µm range, with spectral radiance values averaged over 1 µm intervals.

The dataset was randomly partitioned into training (480,000 samples, 80%), validation (60,000 samples, 10%), and test (60,000 samples, 10%) sets using a fixed random seed (seed = 42) to ensure reproducibility. Stratified sampling was employed to guarantee a uniform distribution of samples across the entire temperature (300–1500 K) and spectral (1–25 µm) ranges.

The fully connected neural network (FCNN) architecture consisted of an input layer (588 neurons), five hidden layers (200 neurons each), and an output layer (24 neurons) [46]. The Adam optimizer was configured with an initial learning rate of 0.001, *β*_1_ = 0.9, *β*_2_ = 0.99, *ε* = 10^–8^, a weight decay of 0.01, and a batch size of 128. ReLU activation was used for the input and output layers, while Sigmoid activation was applied to the hidden layers. The mean squared error (MSE) served as the loss function. With 363,824 trainable parameters, the network was trained on the 480,000-sample training set for 1,200 epochs.

### Computational Setup for the Pareto Solution Set

The normalization formulas for $\mathrm{FOM}$, ${\mathrm{FOM}}_{1}$, ${\mathrm{FOM}}_{2}$, and ${\mathrm{FOM}}_{3}$ are provided below:

2 $$\mathrm{FOM}={w}_{1}\times {\mathrm{FOM}}_{1}+{w}_{2}\times {\mathrm{FOM}}_{2}+{w}_{3}\times {\mathrm{FOM}}_{3}$$

3 $$\begin{aligned} {\mathrm{FOM}}_{1} & = {\mathrm{exp}}( - 0.5 \times \left( {1 - {\mathrm{Spc}}_{{3 - 5}} } \right) \\ & \quad - 0.3 \times {\mathrm{Spc}}_{{5 - 8}} - 0.2 \times \left( {1 - {\mathrm{Spc}}_{{8 - 14}} } \right)) \\ \end{aligned}$$

4 $${\mathrm{FOM}}_{2}=\mathrm{exp}(-\mathrm{Thic}/15600)$$

5 $${\mathrm{FOM}}_{3}=\mathrm{exp}(-\mathrm{Lays}/12)$$

In Eq. (2), the weighting factors ${w}_{1}$, ${w}_{2}$, and ${w}_{3}$ satisfy the constraint ${w}_{1}+{w}_{2}+{w}_{3}=1$, representing the relative contributions of ${\mathrm{FOM}}_{1}$, ${\mathrm{FOM}}_{2}$, and ${\mathrm{FOM}}_{3}$, respectively. Equation (3) defines ${\mathrm{Spc}}_{3-5}$, ${\mathrm{Spc}}_{5-8}$, and ${\mathrm{Spc}}_{8-14}$ as the spectrally integrated average reflectivity of each solution over the 3–5, 5–8, and 8–14 μm infrared bands, respectively. These values are normalized to the range [0, 1]. Equation (4) introduces $\mathrm{Thic}$, which denotes the total thickness of each multilayer solution. The value 15,600 nm represents the maximum allowable thickness threshold, defined by 12 layers each with a upper thickness limit of 1300 nm. In Eq. (5), $\mathrm{Lays}$ refers to the total number of material layers in each solution, with 12 being the predefined maximum number of layers allowed. The values for the aforementioned ${\mathrm{FOM}}_{1}$ to ${\mathrm{FOM}}_{3}$ were all normalized to a range of [e^−1^, 1].

### Infrared Signature and Atmospheric Transmission Modeling

The quantification of infrared signatures and their atmospheric attenuation was performed through an integrated methodology combining fundamental radiation laws with atmospheric radiative transfer modeling.

The inherent radiant properties of the high-temperature sample were characterized using Planck’s law to define its spectral radiance, expressed as Eq. (6):

6 $$B\left(\lambda , T\right)=\frac{2\pi h{c}^{2}}{{\lambda }^{5}}\frac{1}{\mathrm{exp}(hc/\lambda kT)-1}$$

where $B\left(\lambda , T\right)$ denotes the spectral radiance of a black body (W m^−2^ μm^−1^), h is the Planck constant, c represents the speed of light, k is the Boltzmann constant, λ is the wavelength, and T is the absolute temperature in Kelvin.

And the Stefan–Boltzmann law to determine the total radiant exitance, expressed as Eq. (7):

7 $${P}_{\mathrm{rad}}=\epsilon \sigma {T}^{4}$$

where ϵ represents the sample’s average emissivity, and σ is the Stefan–Boltzmann constant (5.67 × 10^–8^ W m^−2^ K^−4^).

To account for atmospheric effects, the MODTRAN® 6 [50, 51] radiative transfer code was employed to simulate key atmospheric parameters across a matrix of environmental conditions. This approach provided the critical spectral atmospheric transmittance, ${\tau }_{\mathrm{atm}}(\lambda )$, and the spectral path radiance, ${L}_{\mathrm{path}}(\lambda )$.

The spectral radiance incident upon the detector, ${L}_{\mathrm{det}}(\lambda )$, was subsequently calculated by coupling the sample’s emission with the atmospheric parameters. The governing equation for this calculation is given by Eq. (8):

8 $${L}_{\mathrm{det}}\left(\epsilon , \lambda \right)=\epsilon \left(\lambda \right)B\left(\lambda , T\right){\tau }_{\mathrm{atm}}\left(\lambda \right)+{L}_{\mathrm{path}}(\lambda )$$

where $\epsilon \left(\lambda \right)$ is the spectral emissivity of the sample, and $B\left(\lambda , T\right)$ is the Planck blackbody spectral radiance.

Finally, to quantitatively assess the suppressive effect of the select radiation design on the target signal, two metrics were computed over the spectral band of interest $[{\lambda }_{1},{\lambda }_{2}]$. The Radiative Suppression Percentage, η, is defined as Eq. (9):

9 $$\eta =\left[1-\frac{{\int }_{{\lambda }_{1}}^{{\lambda }_{2}}{L}_{\mathrm{det}}\left({\epsilon }_{1}, \lambda \right)d\lambda }{{\int }_{{\lambda }_{1}}^{{\lambda }_{2}}{L}_{\mathrm{det}}\left({\epsilon }_{2}, \lambda \right)d\lambda }\right]\times 100\%$$

where ${\epsilon }_{1}$ and ${\epsilon }_{2}$ represent the spectral emissivities of the samples with and without the spectrally selective radiative effect, respectively.

The radiation attenuation in decibels, ${I}_{\mathrm{dB}}$, a logarithmic measure of signal reduction, is calculated as Eq. (10):

10 $${I}_{\mathrm{dB}}=10{\mathrm{log}}_{10}(1-\eta )$$

### Temperature-Dependent Infrared Engineering Data for Each Solution

**Fig. 1:**
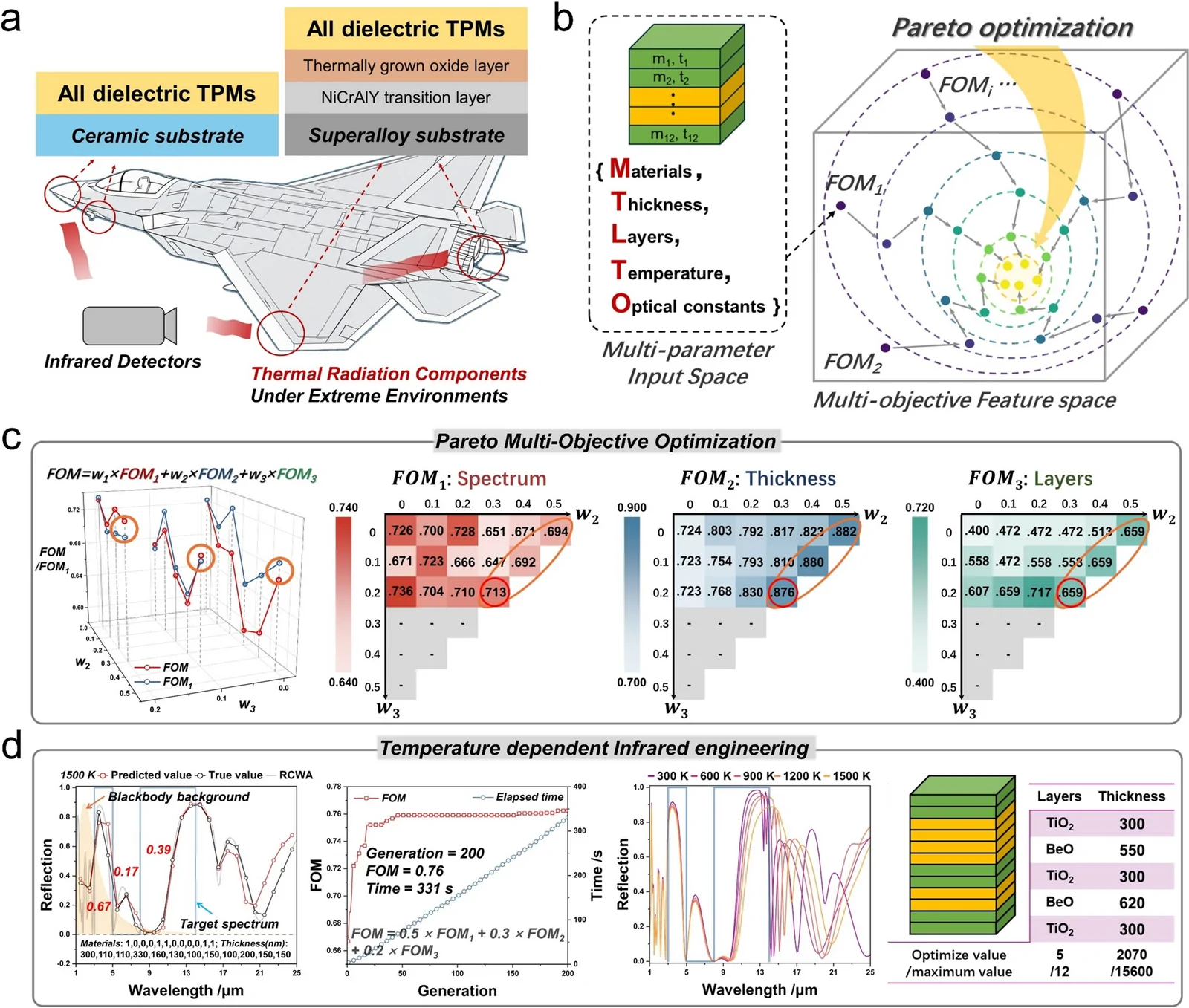
Temperature-dependent infrared engineering framework for all-dielectric TPMs. **a** Device architecture of TPMs integrated onto hot-section components of high-speed aerospace vehicles. **b** Schematic representation of Pareto optimization within the multi-objective feature space. **c** Pareto solution set heatmaps. **d** Computational efficiency and resulting design performance of the temperature-dependent infrared engineering framework

Figures S1-S15 present detailed data for each solution within the Pareto solution set, where the samples are labeled according to their positional coordinates in the w_2_-w_3_ weighting space as illustrated in Fig. 1c. The figure comprises the predicted values from the temperature-dependent neural network, the true values, and the rigorous coupled-wave analysis (RCWA) simulation results for each solution at 1500 K; the evolution of the FOM over 200 generations; the computational time recorded by the algorithm timer; temperature-dependent infrared spectra across the 300–1500 K range; and detailed material-structure sequences, spectral numerical values, number of material layers, and total thickness.

### Film Deposition and Post-Processing

The trade-offs in material properties among candidate high-temperature dielectric layers are summarized in Table S1. From the five materials evaluated, BeO and TiO_2_ were ultimately selected for experimental validation owing to their high melting points, notable refractive index (*n*) contrast, closely matched thermal expansion coefficients (CTE), and excellent high-temperature chemical stability. Moreover, BeO possesses an exceptionally high infrared reflectivity (~ 0.7 in 8–14 μm)—a characteristic distinct from the other candidates—which provides an inherent advantage for designing dual-band stealth metamaterials functional across both the 3–5 and 8–14 μm spectral regions.

The C-axis-oriented sapphire substrates (20 mm × 20 mm × 0.5 mm; Φ100 mm × 0.5 mm; 22.9 mm × 10.2 mm × 3 mm) were ultrasonically cleaned in acetone, ethanol, and deionized water for 15 min each, followed by drying with a nitrogen gun before use. Both BeO and TiO_2_ thin films were deposited via radio frequency (RF) reactive sputtering. BeO films were deposited via reactive magnetron sputtering using high-purity Be targets (Φ100 mm × 2 mm, 99.5 wt.%, Hefei KJ Materials Technology Co., Ltd.) and high-purity argon and oxygen gases (99.999 at%) under optimized conditions: Ar/O₂ flow ratio of 27: 1.5 sccm, sputtering pressure of 0.2 Pa, RF power of 300 W, and deposition rate of 7.2 nm min^−1^. TiO_2_ films were synthesized by reactive magnetron sputtering with stoichiometric TiO_2_ targets (Φ100 mm × 5 mm, 99.9 wt%, Hefei KJ Materials Technology Co., Ltd.) at an Ar/O₂ flow ratio of 24:6 sccm, sputtering pressure of 0.4 Pa, RF power of 300 W, and growth rate of 1.8 nm min^−1^. It should be noted that during the deposition of both BeO and TiO_2_, the substrate temperature was maintained at 800 K.

The Ni-based superalloy substrates (100 mm × 15 mm × 75 μm) were polished, then ultrasonically in acetone, ethyl alcohol and distilled water for 15 min consequently. The substrates were dried with nitrogen gas flow before the deposition. NiCrAlY transition layer film was deposited on the substrate using direct current (DC) magnetron sputtering with high-purity NiCrAlY alloy target (Ni_67_Cr_22_Al_10_Y_1_, 178 mm × 89 mm × 5 mm, 99.99 wt%). After sputtering for 8 h, the thickness of NiCrAlY film reached 15 µm. Then, it was vacuum annealed at 1050 °C for 6 h so that aluminum segregated to the surface of the NiCrAlY film. This NiCrAlY film was further annealed in oxygen at 1050 °C for 6 h to form the thermally grown oxide layer.

Safety protocols for beryllium oxide handling: Due to the known toxicity of BeO particulates, all fabrication and handling procedures were conducted under stringent safety protocols. All handling and deposition processes involving the BeO target were conducted in a dedicated cleanroom facility. This environment was critical not only for guaranteeing the film quality but also for providing the necessary infrastructure to implement rigorous safety measures, including controlled airflow and HEPA filtration, thereby mitigating any potential exposure risks. Personnel handling the BeO target or performing chamber maintenance wore appropriate personal protective equipment, including powered air-purifying respirators (PAPR). All waste materials in contact with BeO were collected and disposed of as hazardous chemical waste in strict accordance with institutional environmental health and safety regulations. It is noted that post-deposition, the BeO is encapsulated within a dense, stable ceramic multilayer structure, significantly mitigating any hazard during subsequent handling and testing.

### Testing and Characterization

The films were prepared by magnetron sputtering (JGP-560, SKY Technology Development Co., Ltd.). Infrared spectrum of the films was, respectively, obtained by the IR Fourier spectrometer (VERTEX80, Bruker) and the integrating sphere system (Nicolet iS50, Thermo Fisher) in the range of 3–14 um. To accurately measure the transmittance (S_21_ magnitude) of TPMs within the 8–12 GHz frequency range and 300–1000 K temperature range using a rectangular waveguide setup and the Vector Network Analyzer (Keysight E5071C/E5063A ENA Series) and a dedicated temperature control unit with an accuracy of ± 3 K. Infrared thermographic characterization of the films was performed using an infrared thermal camera (SPARK M200, TEIOPS) operating within the 3–5 μm spectral band and a temperature range of 458–1709 K, with the atmospheric background radiation set to zero. Scanning electron microscope (JSM-7000F, JEOL) was applied to examine the morphology and microstructure of the films. The crystal structure of the films was examined by X-ray diffraction (XRD, Rigaku D/MAX-rA diffractometer) with the scan range from 20° to 80°. Chemical bonding configurations and chemical states of the sample were revealed by X-ray photoelectron spectroscopy (XPS, Kratos XSAM 800, Al K_α_ radiation). The mechanical properties of the films were carried out via the Nanoindenter (Agilent U9820A Nano Indenter G200) with a diamond Berkovich tip, and the Nanoscratch instrument (Bruker/TI 980). Temperature was monitored using direct and indirect applications of thermocouples. Sample surface temperature was measured with Type-K (NiCr-NiSi) thermocouples, operating from 300 to 1400 K with a tolerance of ± 0.75%. Meanwhile, the high-temperature muffle furnace was controlled using Type-S (PtRh10-Pt) and Type-B (PtRh30-Pt) thermocouples, which cover a range from 300 to 2000 K with a higher accuracy of ± 0.25%.

## Results and Discussion

### Temperature-Dependent Infrared Engineering for All-Dielectric TPMs

Figure 1a illustrates the critical thermal regions of a hypersonic vehicle that exhibit constrained infrared signature characteristics. The selection of substrate materials for TPM fabrication was based on their relevance to key aerospace components. These include single-crystal sapphire, representing optical-grade applications such as sensor windows, and nickel-based superalloys, representing thermo-structural components like engine blades and liners. To enable the in situ integration of all-dielectric multilayer TPMs onto such superalloy substrates, it is essential to first address the severe thermal expansion mismatch at elevated temperatures between the superalloy substrate and the dielectric coating—their coefficients of thermal expansion differ by an order of magnitude. To mitigate this issue, we adopt a thermally grown oxidation process commonly used in thermal barrier coatings (TBCs) and thin-film sensors for aero-engine hot-section components [52]. This approach results in a layered architecture consisting of (from bottom to top): a nickel-based superalloy substrate, a homologous NiCrAlY transition layer, a thermally grown oxide (TGO) interlayer, and the all-dielectric TPMs. Through sequential high-temperature vacuum heat treatment and low-oxygen partial pressure oxidation of the homologous NiCrAlY transition layer, a dense and thermally stable TGO interlayer is formed, achieving a functionally graded transition between the metal and ceramic interfaces.

Figure 1b illustrates the Pareto optimization approach within a multi-objective feature space for temperature-dependent infrared engineering. The proposed framework builds upon a previously established single-objective algorithm, incorporating a temperature-dependent phonon self-energy dataset and a dimensionality-augmented neural network [46]. This enhanced architecture enables rapid inverse design—achieving a 120-fold acceleration compared to conventional analytical algorithm—across a broad thermal range (300–1500 K) and the full infrared spectrum (1–25 μm), while maintaining high generalizability. To address extreme operational environments exceeding 1500 K for infrared stealth applications, we further refined the algorithm by integrating critical practical constraints, including ambient temperature, thermal stress compatibility, film thickness, and metamaterial layer count. This multi-objective optimization framework significantly improves the adaptability and robustness of the temperature-adaptive neural network, enhancing its suitability for real-world infrared stealth engineering scenarios.

Figure 1c presents the Pareto solution set heatmaps derived from multi-objective optimization incorporating infrared spectral performance (FOM_1_), total metamaterial thickness (FOM_2_), and number of thin-film layers (FOM_3_). All figures of merit (FOM_i_) and their corresponding weighting factors (w_i_) were normalized prior to analysis; the detailed computational procedures are provided in the Methods. Notably, when w_3_ ≥ 0.3, the composite FOM overwhelmingly favors minimal layer configurations—often converging to a single-layer structure—resulting in overfitting and poor generalizability. Therefore, data corresponding to w_3_ ≥ 0.3 are excluded from the table. Based on their coordinates within the w_2_–w_3_ parameter space, the samples are labeled from S_00_ to S_32_, totaling 15 specimens. The corresponding detailed computational results are provided in Figs. S1–S15. Table S2 presents the root mean square error (RMSE) between the GA-NN algorithm and the RCWA for samples S00 to S32, accompanied by error visualization heatmaps. The data indicate that four sample groups—S10, S30, S01, and S21—exhibit relatively high reflectance errors (0.12–0.19), corresponding to structures with 7–9 layers and total thicknesses ranging from 3160 to 5060 nm (Figs. S1-S15). In contrast, samples S50, S41, S22, and S32 show lower reflectance errors (0.04–0.13), corresponding to structures with 5–6 layers and thicknesses between 1990 and 2900 nm. These results demonstrate that the GA-NN algorithm achieves higher accuracy in generating solutions with fewer layers and smaller thicknesses, which aligns well with the design objectives for TPMs in extreme environments that prioritize component lightweighting and structural efficiency. Furthermore, Table S3 provides a quantitative comparison between the temperature-dependent infrared engineering model and a baseline model. The results reveal that as the temperature increases to 1500 K, the temperature-induced reflectance error can reach up to 0.24, clearly underscoring the necessity of employing temperature-dependent infrared engineering in the design of TPMs for extreme environments.

A summary of the computational results for the solution set is provided in Table S4, with the top five values in each column highlighted in bold and underlined. Samples S_00_, S_20_, and S_02_ exhibit the highest weighting for infrared spectral performance (FOM_1_), yielding superior spectral values (0.726, 0.728, and 0.736, respectively). However, they demonstrate moderate performance in total thickness (FOM_2_; 0.724, 0.792, and 0.722) and notably poor results in layer count (FOM_3_; 0.400, 0.472, and 0.607). Among the 15 samples, S_02_, S_22_, and S_32_ exhibit the best comprehensive FOM, with values of 0.734, 0.735, and 0.730, respectively. Notably, S_32_ also demonstrates well-balanced performance across FOM_1_, FOM_2_, and FOM_3_ (0.713, 0.878, and 0.659, respectively). Therefore, S_32_ was selected for subsequent computational and experimental validation owing to its optimal trade-off among all performance metrics.

Figure 1d presents the temperature-dependent infrared engineering design data for sample S_32_. The comparison between the GA-NN algorithm and the RCWA method across the 1–25 μm infrared range at 1500 K is displayed, where the predicted values (from GA-NN) and ground-truth values (from RCWA) show excellent agreement, particularly within the target spectral region of 3–14 μm (Table S3). The reflectivity values of S_32_ in the 3–5, 5–8, and 8–14 μm bands are 0.67, 0.17, and 0.39, respectively. Targeting infrared stealth under extreme conditions, the upper limit of our theoretical spectral analysis, 1500 K, was selected as the TPM design benchmark. The band-integrated radiative (BIR) power was calculated at 1500 K, revealing that the 3–5, 5–8, and 8–14 μm bands contribute 0.64, 0.26, and 0.10 to the total power, respectively. However, assigning a weight of 0.1 to the 8–14 μm band caused FOM_1_ to be overwhelmingly dominated by the 3–5 and 5–8 μm bands. This led to overfitting and poor generalizability, thereby compromising the design for dual-band infrared stealth. Therefore, through comprehensive consideration of both the blackbody radiation distribution and the optimization objectives, the weighting factors for these three bands in FOM_1_ were assigned as 0.5, 0.3, and 0.2, respectively. Further details regarding the composite figure of merit (FOM) configuration are provided in the Supplementary Information.

The evolution of the FOM over 200 generations (with a population size of 200 per generation) is illustrated along with a computational timer. The FOM reaches its maximum value and stabilizes after 100 generations. As shown in Fig. S16, at the 100-generation mark, the runtimes of the GA-RCWA, GA-NN, and the multi-objective algorithm developed in this work are 19,321, 160, and 171 s, respectively. This corresponds to a per‑individual computation time per generation of 970, 8, and 8.6 ms, indicating that both the GA-NN and the multi‑objective algorithm exceed the speed of the GA-RCWA by two orders of magnitude. Furthermore, the computation times for the GA-NN and the multi‑objective algorithm at the first generation are 1.9 and 8.5 s, respectively, suggesting that the initial delay of the multi-objective algorithm relative to GA-NN primarily stems from differences in the speed of generating the initial random population, while subsequent optimization efficiency remains comparable. Ultimately, the total optimization time over 200 generations is 362 s, corresponding to a mere 9.1 ms per evaluation. Compared to the conventional RCWA-based approach (9.1 vs. 970 ms per evaluation), this represents a 100-fold speedup, highlighting the computational efficiency of the enhanced GA-NN algorithm for multi-objective optimization. Furthermore, the infrared spectral performance of the all-dielectric TPM is evaluated across a broad temperature range (300–1500 K). The results indicate remarkable spectral stability within the 3–8 μm band, demonstrating minimal temperature-dependent variation. Finally, the material composition and structural parameters of the TPM are provided: The multilayer stack, from top to bottom, consists of TiO_2_/BeO/TiO_2_/BeO/TiO_2_, with individual layer thicknesses of 300, 550, 300, 620, and 300 nm, respectively. The entire structure comprises only 5 layers with a total thickness of 2.07 μm.

**Fig. 2:**
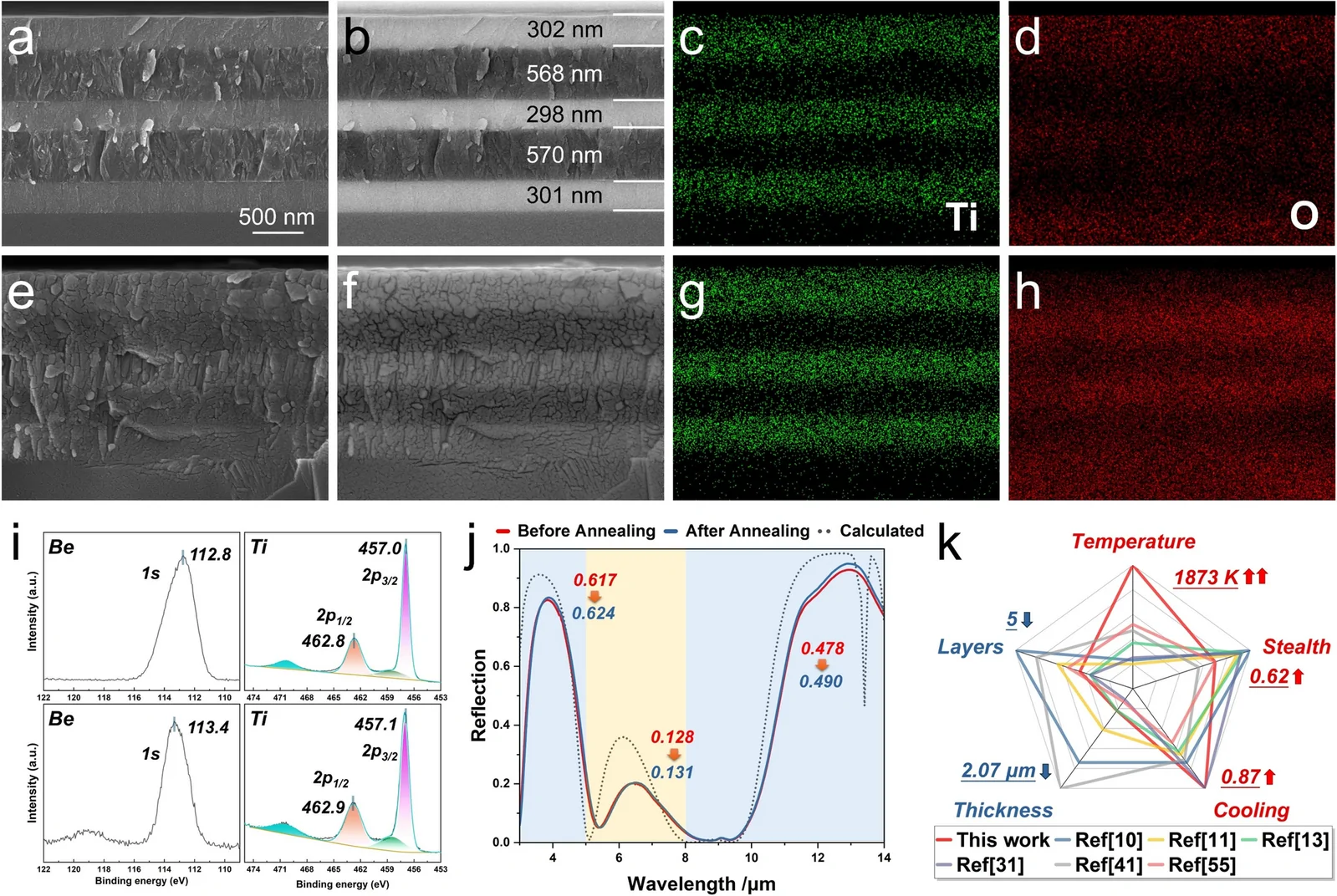
Extreme-temperature thermal stability of all-dielectric TPMs. **a, e** Secondary electron micrographs. **b, f** Backscattered electron images before and after annealing. **c, g** EDS elemental mapping of titanium (Ti). **d, h** EDS elemental mapping of oxygen (O). **i** XPS analysis of BeO and TiO_2_. **j** FTIR reflectance spectra before and after annealing. **k** Radar chart comparing key performance metrics in this work with previously reported systems

Figure S17 presents a schematic diagram of the atmospheric transmission model constructed using MODTRAN® 6 [50, 51], which was employed to systematically investigate the radiative suppression performance of sample S32 under various atmospheric conditions. Three representative atmospheric models—Mid-Latitude Summer (MLS), Tropical (TROP), and Sub-Arctic Winter (SAW)—were employed under clear-sky conditions with 23 km visibility. The simulations were performed using default gas concentration profiles (CO_2_ at 400 ppm, H_2_O vapor as per model specification). The simulation covered target altitudes of 1, 5, and 15 km, with the sensor altitude fixed at 20 km to calculate slant-path lengths. The sensor bandwidth was set to the 3–5 μm infrared detection window, assuming optical components at a low space-background temperature (self-emission neglected). The emissivity inputs were: the experimentally measured ε(λ) of the TPM sample (Fig. 2j), a constant ε = 0.8 for the oxidized alloy reference substrate. Figure S18 displays the corresponding atmospheric transmittance and background radiance spectra for all nine simulated scenarios. Figures S19-S21 comparatively present the detected spectral radiance of sample S32 against a metal oxide substrate (emissivity set to 0.8) across a temperature range of 300–1500 K for the nine simulation settings, respectively. Table S5 summarizes the band-integrated radiance within the 3–5 µm infrared detection window for both sample S32 and the oxide substrate across the same temperature range and scenarios. Table S6 quantitatively lists the corresponding radiative suppression efficiency and attenuation values. Analysis of Table S6 reveals that sample S32 exhibits its weakest radiative suppression performance in the Sub-Arctic Winter model, where atmospheric transmittance is highest. Excluding the atmospheric background radiation at 300 K, the sample demonstrates a minimum suppression efficiency of 51% and an attenuation of − 3.1 dB at a low temperature of 600 K. As the sample temperature increases to 1200–1500 K, the suppression efficiency improves significantly, reaching a maximum of 70% with an attenuation of − 5.2 dB. In contrast, under the lower-transmittance conditions of the Mid-Latitude Summer and Tropical models, sample S32 achieves superior radiative suppression. At 600 K, the minimum suppression efficiency remains as high as 64% with an attenuation of − 4.7 dB. When the temperature rises to 1200–1500 K, the suppression efficiency peaks at 82%, accompanied by a strong attenuation of − 7.4 dB.

### Extreme-Temperature Thermal Stability of All-Dielectric TPMs

Figure 2 demonstrates the extreme-temperature thermal stability of the all-dielectric TPM—fabricated according to the design in Fig. 1d—in terms of its microstructure, physicochemical properties, and infrared spectral performance. The TPM was deposited via magnetron sputtering; detailed fabrication procedures are provided in the Methods.

Figure 2a, b shows cross-sectional secondary electron (SE) and backscattered electron (BSE) images of the as-deposited TPM, respectively. Both images reveal distinct layering between TiO_2_ and BeO films. The TiO_2_ layers consist of finely packed small crystallites, while the BeO layers exhibit relatively coarse columnar grains. Thickness measurements from Fig. 2b indicate individual layers of approximately 302/568/298/570/301 nm, within 5% deviation from the designed values. Figure 2c, d presents energy-dispersive spectroscopy (EDS) elemental maps. Ti is concentrated in the 1st, 3rd, and 5th layers (TiO_2_), while O is uniformly distributed across both TiO_2_ and BeO layers. Note that Be signals are barely detectable due to its low atomic mass.

After annealing at 1873 K in air for 12 h, the SE image in Fig. 2e shows that the layered structure becomes less distinct. Both TiO_2_ and BeO have undergone Ostwald ripening, forming larger equiaxed grains. The overall structure remains intact with strong interlayer adhesion and no visible cracking, despite the presence of high-angle grain boundaries. The BSE image in Fig. 2f, however, still clearly reveals the five-layer periodic structure with unchanged layer thicknesses, confirming exceptional structural stability under extreme temperatures. EDS mapping in Fig. 2g, h continues to show Ti localization in the original TiO_2_ layers and uniform oxygen distribution, indicating the absence of interdiffusion or solid-solution reactions between TiO_2_ and BeO at high temperature.

X-ray photoelectron spectroscopy (XPS) results before and after annealing are shown in Fig. 2i. The Be 1*s* peak shifts from 112.8 to 113.4 eV, suggesting improved crystallinity of BeO—consistent with the transition from columnar to equiaxed grains [53]. The Ti 2*p* peaks (2*p*_3/2_ and 2*p*_1/2_) shift slightly by 0.1 eV to higher binding energies (457.1 and 462.9 eV, respectively), likely due to crystallization or minor lattice distortion [54]. The constant spin-energy separation of 5.8 eV between Ti 2*p* peaks confirms the typical TiO_2_ bonding state. Additionally, the X-ray diffraction (XRD) results indicate that both BeO and TiO_2_ exhibit sharp, well-defined crystalline peaks after annealing, as shown in Fig. S22. This presents a marked contrast to the broad, amorphous peaks (or absence of distinct peaks) observed in the as-deposited state prior to annealing. Figure 2j compares experimental and simulated infrared reflectance spectra before and after annealing. The infrared reflectance spectra were acquired using a Fourier-transform infrared (FTIR) spectrometer. The as-fabricated TPM exhibits reflectances of 0.617/0.128/0.478 in the 3–5/5–8/8–14 μm bands, consistent with simulations (0.67/0.17/0.39). Post-annealing reflectances change marginally to 0.624/0.131/0.490—a variation of less than 3%—demonstrating remarkable spectral stability under extreme thermal conditions.

A radar chart in Fig. 2k compares key performance metrics of this work with recently reported multilayer TPMs [10, 11, 13, 31, 41, 55]. The all-dielectric TPM presented here achieves a significant increase in operational temperature (from 1173 to 1873 K) while maintaining high performance: strong infrared camouflage in the 3–5 μm band (reflectance > 0.62) and efficient radiative cooling in the 5–8 μm band (emittance > 0.87). Moreover, it features a minimal number of layers (5) and 2 μm total thickness (2.07 μm). The all-dielectric TPM with a periodic TiO_2_/BeO layered structure retains excellent structural integrity, physicochemical stability, and spectral performance even after 12 h of annealing at 1873 K in air.

### Thermo-Mechanical Robustness Evaluation of All-Dielectric TPMs

**Fig. 3:**
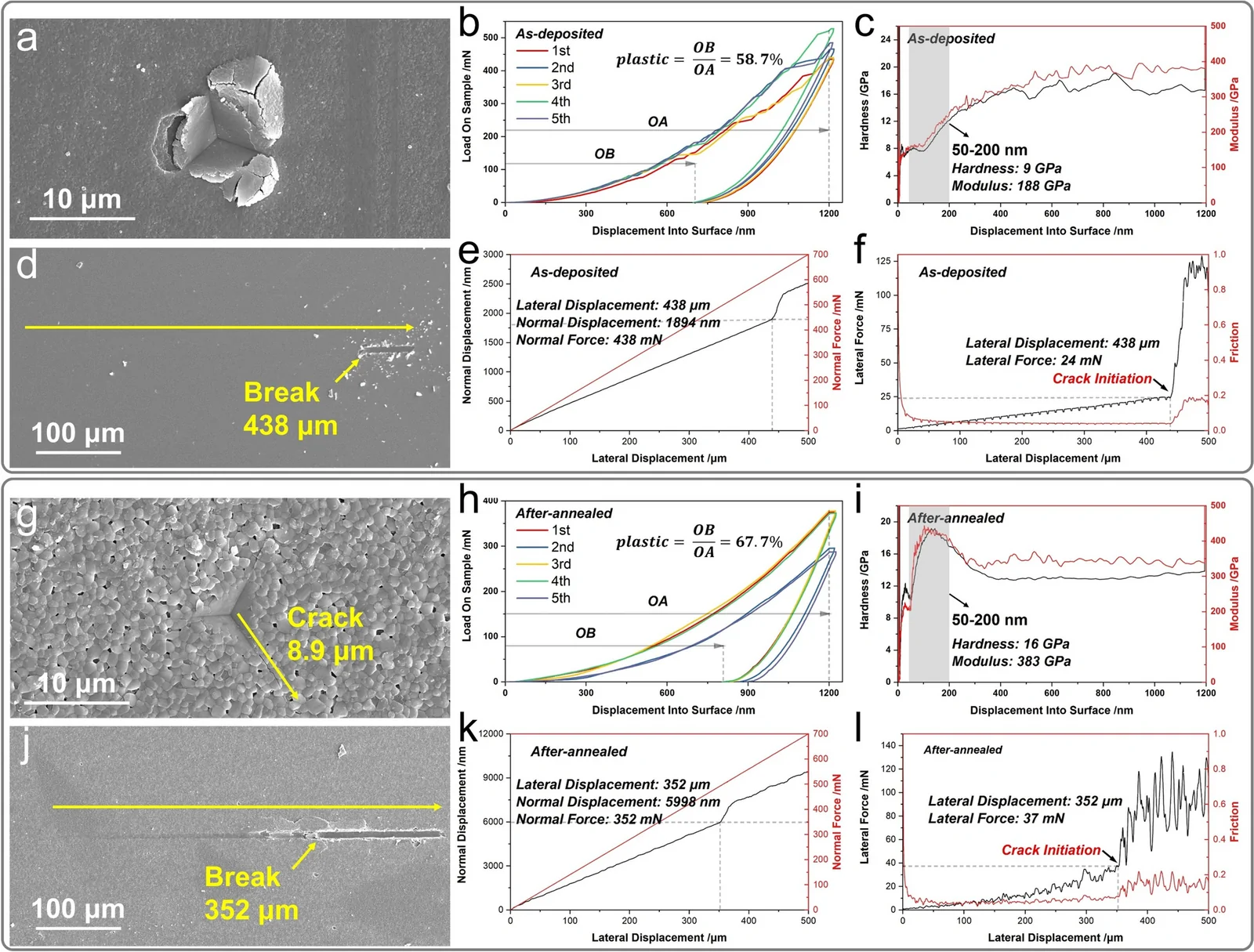
Mechanical properties of all-dielectric TPMs. **a**–**c** Nano-indentation testing before annealing. **d**–**f** Nano-scratch testing before annealing. **g**–**i** Nano-indentation after annealing. **j**–**l** Nano-scratch testing after annealing

Figure 3 presents the thermo-mechanical robustness evaluation of all-dielectric TPMs. Excellent thermal robustness is essential for maintaining the structural integrity and functional performance of TPMs under extreme operational conditions, including high temperatures, high-speed fluid erosion, intense vibration, and severe thermal shocks.

Figure 3a, g displays the nanoindentation morphology of the as-deposited and annealed TPMs, respectively, obtained using a Berkovich indenter at five different locations. At a maximum penetration depth of 1200 nm, Fig. 3a reveals pronounced delamination cracks without observable edge cracking, indicating that fracture energy was absorbed primarily through interlayer decohesion. In contrast, Fig. 3g shows edge cracks with lengths of approximately 8.9 μm, suggesting enhanced interfacial bonding and a transition toward a rigid ceramic-like film after annealing.

Figure 3b, h presents the load–unload curves from five independent nanoindentation tests, demonstrating mechanical homogeneity across the sample. The load curve in Fig. 3b exhibits multiple pop-in events, indicative of stress release through fracture, consistent with the delamination behavior observed in Fig. 3a. At maximum load, the penetration depth reached 1200 nm, with an elastic recovery of 500 nm and a residual depth of 700 nm after unloading, corresponding to a plastic deformation ratio of 58.7% and an elastic contribution of 41.3%. By comparison, the load curve in Fig. 3h shows no significant pop-ins, implying a more homogeneous mechanical response and effective relief of residual interfacial stresses after annealing. Furthermore, the annealed TPM exhibits a plastic deformation ratio of 67.7%, representing a 9% increase compared to the as-deposited state.

Figure 3c, i summarizes the hardness and Young’s modulus of the TPM before and after annealing, averaged over the initial 10% of the total film thickness (200 nm). The as-deposited TPM shows values of 9 GPa and 188 GPa, respectively, which increase to 16 GPa and 383 GPa after annealing. This notable enhancement in hardness aligns with typical characteristics of rigid ceramic films. According to the Anstis model [56], the fracture toughness of the annealed TPM is calculated to be approximately 1.1 MPa m^1/2^. Furthermore, a benchmark yttria-stabilized zirconia (YSZ) film—a commonly used high-temperature thermal barrier coating (TBC) material—of comparable thickness (~ 2 μm) was fabricated for mechanical performance comparison. As shown in Fig. S23, the annealed YSZ film exhibits a fracture toughness of 1.3 MPa m^1/2^, which is marginally higher than the 1.1 MPa m^1/2^ measured for TPM. This result indicates that the developed TPM possess a comparably excellent level of fracture toughness.

Figure 3d, j presents the nanoscratch morphology of the TPM before and after annealing, performed using a diamond tip (radius = 4.7 μm) along three distinct paths. The scratches appear as straight-line features, each not exceeding 500 μm in length. Figure 3e, f shows the corresponding loading curve and the profiles of friction force/coefficient for the as-deposited TPM, respectively. An abrupt change in friction occurs at a scratch length of 438 μm (Fig. 3f), indicating film cracking or interfacial delamination. This corresponds to a penetration depth of 1894 nm and a critical load of 438 mN (Fig. 3e). Figure 3k, l displays the nanoscratch results after annealing, where the friction transition occurs at a shorter scratch length of 352 μm, corresponding to a penetration depth of 5,998 nm and a critical load of 352 mN.

Due to the complexity and practical challenges in accurately determining interfacial adhesion strength through contact mechanics models [57, 58], the critical load derived from scratch testing serves as a qualitative proxy for interfacial bonding strength. The critical load of the annealed TPM is 352 mN, compared to 438 mN for the as-deposited sample, indicating that the interfacial adhesion strength retains more than 80% of its original value after annealing. Under identical conditions, the annealed YSZ exhibited a mechanical performance improvement to 106% of its pre-annealed value, demonstrating excellent interfacial adhesion, as shown in Fig. S23. It is noteworthy that the TPMs maintained 80% of their interfacial adhesion strength, despite their more complex interface morphology and the absence of a phase-transformation toughening mechanism inherent to YSZ.

Following extreme-temperature annealing, the annealed TPMs exhibit an increased ratio of plastic deformation and a significant enhancement in interlayer adhesion strength, indicating a progressive transition toward a rigid ceramic film. Remarkably, the annealed TPMs retain a fracture toughness of 1.1 MPa m^1/2^ and preserve over 80% of their original interfacial adhesion strength compared to the as-deposited state. This performance corresponds to a level of thermo-mechanical robustness that is comparable, albeit slightly lower, to that of the conventional TBC material YSZ.

### High-Temperature Infrared Thermography of All-Dielectric TPMs

**Fig. 4:**
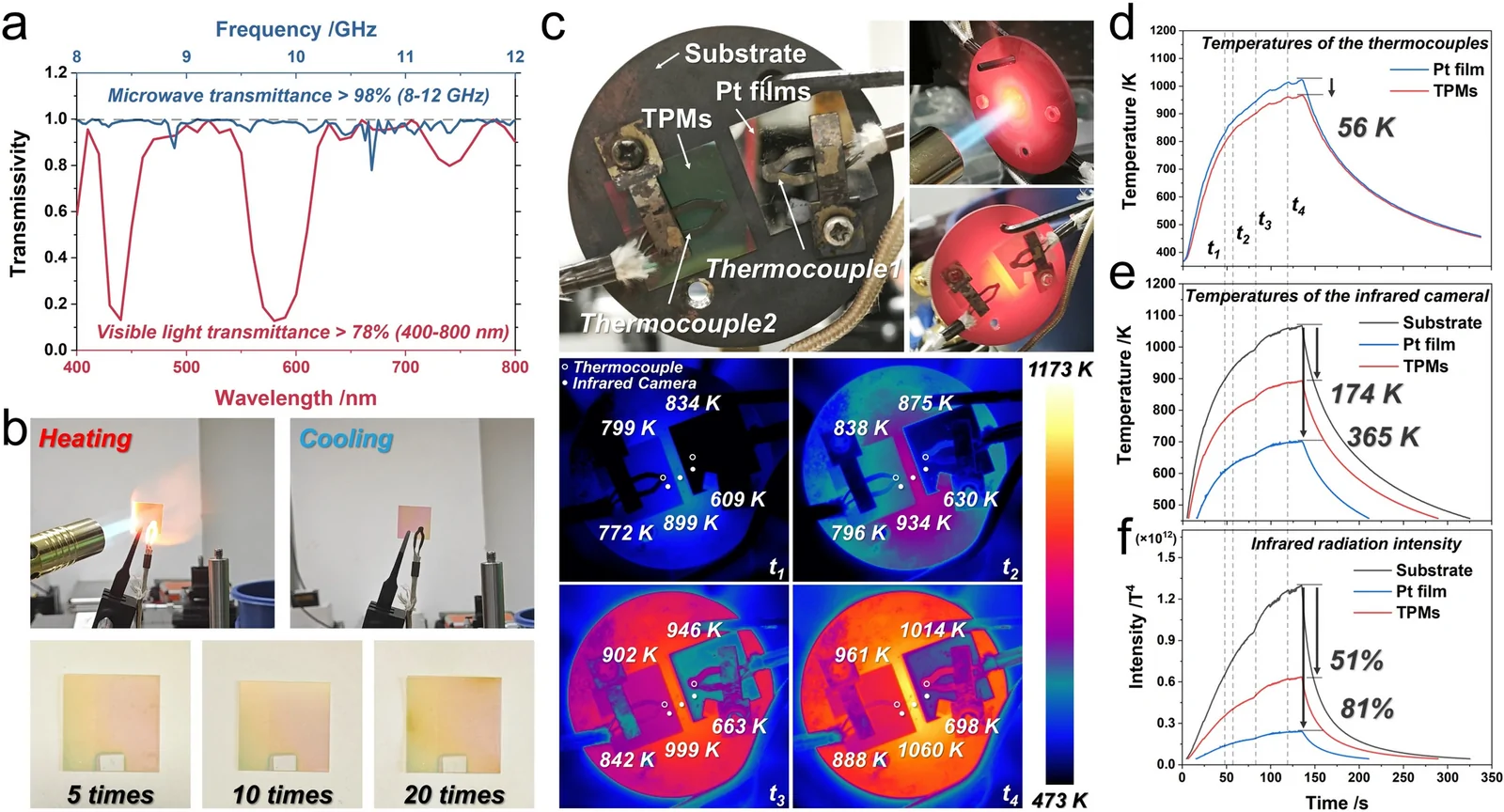
High-temperature infrared performance of all-dielectric TPMs on sapphire substrate. **a** Visible and microwave transmittance. **b** Thermal shock testing. **c** High-temperature infrared thermography. **d**–**f** Radiative suppression and cooling performance

Figure 4 presents an infrared thermographic evaluation of TPMs directly integrated onto a sapphire substrate. The detection window should exhibit not only controllable infrared spectral properties but also good transmittance compatibility in the visible and microwave bands. Figure 4a shows the visible and microwave transmittance of TPMs on sapphire. Due to Fabry–Pérot cavity resonance, reflection or absorption peaks occur at 440 and 580 nm; nevertheless, the structure maintains an average visible transmittance of 78% across the 400–800 nm band. Owing to its all-dielectric material composition and a mere 2 μm thickness, the TPM achieves a microwave transmittance as high as 98% in the X-band (8–12 GHz) at room temperature, as shown in Fig. S24. Furthermore, under high-temperature testing conditions ranging from 300 to 1000 K, the TPM maintains an exceptional transmittance of over 98% across the X-band, as demonstrated in Fig. S25. Prior to infrared thermography, the TPM was subjected to a thermal shock test. The temperature cycled between 700 and 1400 K, with a heating time of 4–5 s (rate of 140–175 K s^−1^) and a cooling time of 10–14 s (rate of 50–70 K s^−1^). The corresponding temperature profiles are detailed in Fig. S26. As shown in Fig. 4b, during 20 consecutive thermal shock cycles with heating rates exceeding 150 K s^−1^, darkening occurred along the edges due to uneven heating and heat transfer limitations, yet no significant cracking or delamination was observed (Video S1).

Figure 4c displays the high-temperature infrared imaging results after thermal shock testing (Video S2). The experimental setup consisted of a nickel-based superalloy stage, a sapphire substrate with the TPM, and a reference sapphire substrate coated with a Pt film. Two surface-contact patch thermocouples were used for real-time temperature monitoring: Thermocouple 1 measured the TPM surface temperature, while Thermocouple 2 recorded that of the Pt film. The sample was heated from the back with a butane flame torch, while infrared imaging was performed on the front. Figure 4c shows transient thermal images captured at four time points (t_1_ to t_4_) during heating. Throughout the process, both the TPM and Pt film consistently exhibited lower surface temperatures than the oxidized alloy substrate (emissivity ~ 0.8). The temperature difference between the TPM and the substrate increased progressively—from 127 to 138, 157 K, and finally 172 K. At the peak substrate temperature of 1060 K, the TPM-coated region registered 888 K, corresponding to a temperature reduction of 172 K, confirming its effective radiative suppression function. Under the same conditions, the ideal low-emissivity Pt film showed an imaging temperature of 698 K, which is 362 K lower than the substrate and 190 K lower than the TPM.

Figure 4d presents real-time temperature profiles obtained from both thermocouples and infrared imaging. The thermocouple data confirm that the TPM consistently remained cooler than the Pt film during active heating. Thermocouple measurements revealed that the TPM surface reached 961 K, whereas the Pt film attained 1014 K at its peak, indicating a passive radiative cooling effect of 53 K inherent to the TPM design. According to the Stefan–Boltzmann law, and neglecting atmospheric background radiation at such extreme temperatures, the radiative power emitted from the TPM surface was calculated to be 10.1 kW m^−2^. This value is consistent with the theoretical radiative cooling efficiency of the 5–8 μm atmospheric window in the 900–1200 K range, as derived in this work and supported by Fig. S26. During the cooling phase, the temperature curves of both regions nearly overlapped, indicating that heat conduction through the sapphire and substrate, along with convective heat exchange, dominated the cooling process. The infrared imaging results further corroborate the persistent cooling effect over the TPM region during heating. Cross-referencing with thermocouple data confirms that this enhanced thermal masking results from the combined effects of passive radiative cooling (via the 5–8 μm band) and radiative suppression in the 3–5 μm atmospheric window.

Since infrared thermography infers temperature from thermal radiation intensity, the radiance of the TPM and Pt film was quantitatively compared. At thermal equilibrium, the TPM exhibited 51% lower radiance than the oxidized alloy substrate. Although this is 30% less than the 81% radiance suppression achieved by the Pt film, it still underscores the TPM’s considerable radiative suppression capability. Consistent with previous findings [46], the TPM on sapphire—a commonly used window material—achieves 51% radiative suppression at 1100 K, only 30% less than the ideal low-emissivity Pt film, while also offering additional functionalities absent in Pt, including radiative cooling (~ 53 K), visible transparency (78%), microwave transparency (98%), thermal shock resistance (> 150 K s^−1^), and extreme high-temperature stability (up to 1873 K in air).

**Fig. 5:**
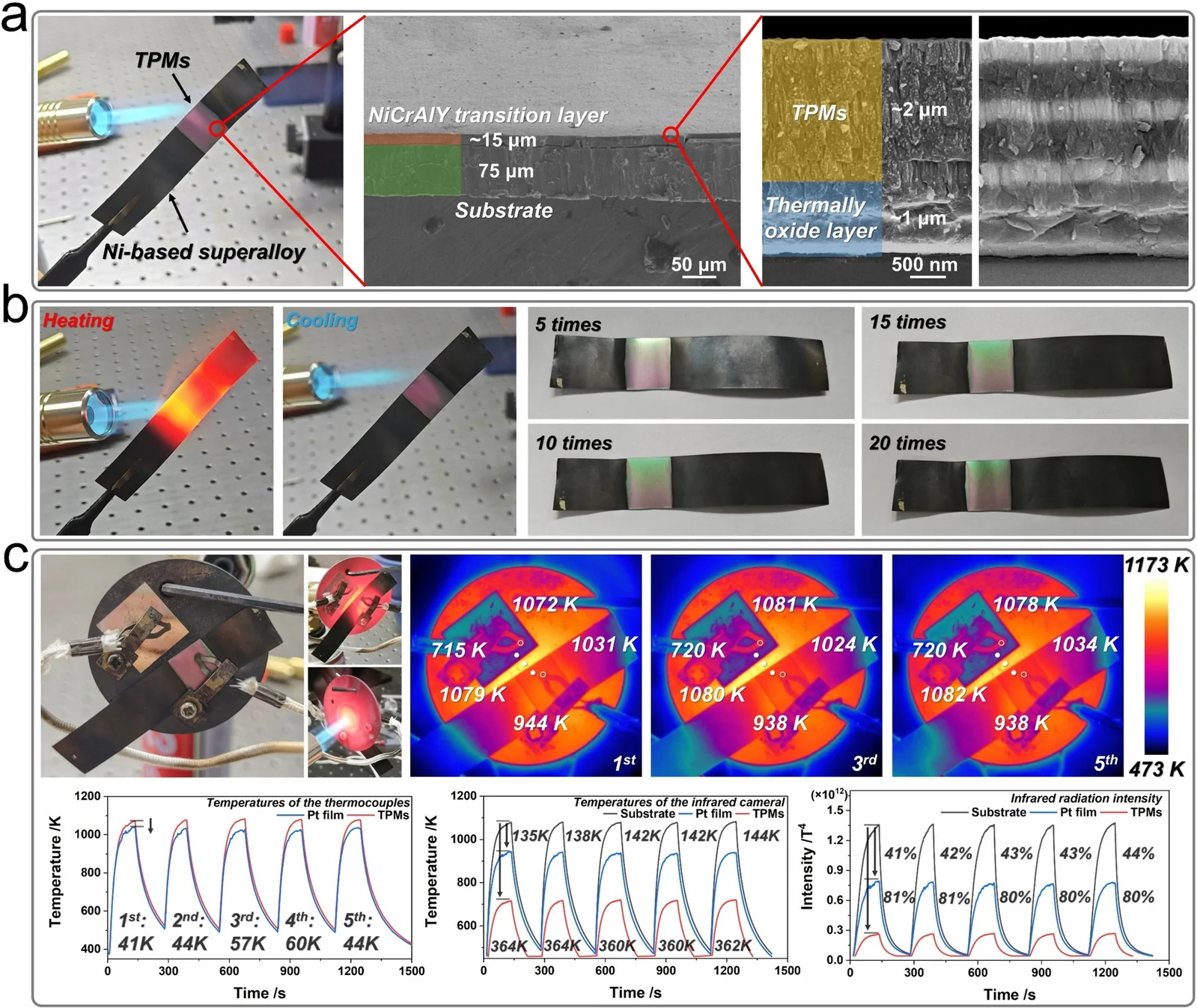
High-temperature infrared performance of all-dielectric TPMs on Ni-based superalloy substrate. **a** Microstructural morphology of the TPMs. **b** Thermal shock testing. **c** Results from five cycles of high-temperature infrared thermography tests

Figure 5 presents an infrared thermographic evaluation of TPMs integrated onto a nickel-based superalloy substrate. In contrast to the requirements for visible and microwave transmittance in optical window applications, the performance stability of TPMs under thermal cycling is of greater concern for hot-section components. Figure 5a displays the cross-sectional morphology of the actual TPM device based on the thermally matched structure illustrated in Fig. 1a. A 15-μm-thick NiCrAlY transition layer was first deposited on a 75-μm-thick superalloy substrate. A thermally grown oxide (TGO) layer was subsequently formed via in situ thermal oxidation, followed by the fabrication of a five-layer TPM structure (SE and BSE images) on top of the TGO layer. Prior to infrared thermography, the TPM underwent thermal shock testing. As shown in Fig. 5b, the TPM surface remained optically intact with no noticeable cracking or delamination after 20 consecutive thermal shock cycles with heating rates exceeding 150 K s^−1^ (see Video S3).

Furthermore, Fig. S28 presents the nanoindentation and nanoscratch test results of the TPM on the superalloy substrate after thermal shock testing. Given that a hard thin film was deposited on a ductile substrate, some inherent variability in the mechanical test results is to be expected. The nanoindentation results indicate that no significant edge cracking occurred in the TPM after thermal shock, suggesting that the fracture threshold was not reached. The nanoscratch results demonstrate that the TPM remained intact without fracture or delamination until the probe penetrated into the NiCrAlY transition layer at a penetration depth of 2566 nm and a scratch length of 305 μm (Fig. 5a). It is noteworthy that, unlike the case in Fig. 3, the coefficient of friction and the frictional force during the scratching of the TPM on the superalloy substrate showed no abrupt jumps. Instead, they increased gradually only after the probe entered the NiCrAlY layer, correlating with the increasing scratch depth. Both the nanoindentation and nanoscratch results confirm that the introduction of the TGO layer on the superalloy substrate significantly enhances the fracture toughness and interfacial adhesion strength of the TPM compared to its previous implementation on a rigid sapphire substrate. This finding is consistent with the thermo-mechanical performance observed in our prior work involving multilayer temperature sensors fabricated on nickel-based superalloy rotor blades and Hastelloy substrates [44, 45].

Meanwhile, following the thermal shock test, infrared imaging experiments analogous to those in Fig. 4c were conducted over five consecutive thermal cycles between high and low temperatures (Video S4). Transient thermal images captured at the maximum temperature during the 1st, 3rd, and 5th cycles are presented. The TPM fabricated on the Ni-based superalloy demonstrates excellent performance, combining substantial radiative suppression and cooling with remarkable reliability. Specifically, it achieved a temperature reduction of 135–144 K (41%–44% suppression) due to its low emissivity (0.38 vs. ~ 0.7 for the oxidized alloy in the 3–5 μm band; Fig. 2j), and a radiative cooling effect of 41–60 K, corresponding to a radiant cooling power of ~ 10.1 kW m^−2^, which aligns with theoretical predictions for the 5–8 μm window (Fig. S27). Critically, this performance remained stable over repeated thermal cycles. These experimental findings provide valuable validation data for the in situ integration of TPMs onto hot-section components of aerospace vehicles.

## Conclusions

In this work, we have demonstrated a holistic temperature-dependent infrared engineering strategy for the design and fabrication of all-dielectric TPMs that exhibit exceptional performance and stability under extreme thermal conditions. By integrating a Pareto multi-objective optimization framework with a temperature-adaptive neural network, we successfully co-optimized optical performance, structural efficiency, and thermal robustness, achieving a computational speed 100 times faster than conventional genetic algorithms. The resulting TPM, composed of a refractory TiO_2_/BeO multilayer stack with only 5 layers and a total thickness of 2.07 μm, exhibits high reflectivity in the 3–5 and 8–14 μm bands (0.62 and 0.48, respectively) for radiative suppression, coupled with high emissivity (0.87) in the 5–8 μm band for efficient radiative cooling. The performance of the TPM was evaluated under various atmospheric models and detection distances. The TPM achieved a peak radiance suppression efficiency of 82% and a maximum attenuation of − 7.4 dB at 1200–1500 K.

Remarkably, the fabricated TPM maintains excellent structural integrity and spectral stability after prolonged exposure to 1873 K in air for 12 h, with less than 3% spectral drift. It also demonstrates outstanding mechanical properties, including high fracture toughness (1.1 MPa m^1/2^) and retained interfacial adhesion strength (> 80%). When deployed on aerospace-grade substrates, the TPM achieves 40%–50% radiative suppression and 40–60 K (~ 10.1 kW m^−2^) passive cooling at 1100 K, alongside high transmittance in both visible (78%) and microwave (98%) bands, ensuring multispectral compatibility. Furthermore, the material withstands severe thermal shocks (> 20 cycles at heating rates exceeding 150 K s^−1^) and multiple thermal cycles without performance degradation. This study establishes a new paradigm for the design and application of photonic materials in extreme environments, offering a scalable and efficient pathway toward high-performance, multispectral functional materials for next-generation aerospace and energy systems.

## Supplementary Information

Below is the link to the electronic supplementary material.

Supplementary file1 (MP4 4586 kb)

Supplementary file2 (MP4 4125 kb)

Supplementary file3 (MP4 6800 kb)

Supplementary file4 (MP4 3631 kb)

Supplementary file5 (DOCX 7365 kb)
