# Supplementary material for: Temperature-Dependent Infrared Engineering for Extreme Environments: All-Dielectric Thermal Photonic Metamaterials Stable at 1873 K in Air
Source: Nanomicro Lett. 2026 Jan 21;18:214. doi: 10.1007/s40820-025-02065-9 (PMC12824092; doi:10.1007/s40820-025-02065-9)
Supplement: Supplementary file 5 — Supplementary file5 (DOCX 7365 kb) [file 40820_2025_2065_MOESM5_ESM.docx]

Supporting Information for

**Temperature-Dependent Infrared Engineering for Extreme Environments: All-Dielectric** **Thermal Photonic Metamaterials Stable at 1873 K in Air**

Yang Liu^1#^, He Lin^1,2,3#^, Yunxia Zhou^1#^, Liming Yuan^1^, Yanqin Wang^1,2^, Xiaoliang Ma^1,2^, Cheng Huang^1,2^, Xiangang Luo^1,2^

*^1^*State Key Laboratory of Optical Field Manipulation Science and Technology, Institute of Optics and Electronics, Chinese Academy of Sciences, Chengdu 610209, P. R. China

*^2^*College of Materials Sciences and Opto-Electronic Technology, University of Chinese Academy of Sciences, Beijing 100049, P. R. China

*^3^*School of Automation Engineering, University of Electronic Science and Technology of China, Chengdu 611731, P. R. China

#Yang Liu, He Lin, and Yunxia Zhou contributed equally to this work.

*Corresponding authors. E-mail: [maxl@ioe.ac.cn](mailto:maxl@ioe.ac.cn) (Xiaoliang Ma); [huangc@ioe.ac.cn](mailto:huangc@ioe.ac.cn) (Cheng Huang); [lxg@ioe.ac.cn](mailto:lxg@ioe.ac.cn) (Xiangang Luo)

**Supplementary Figures and Tables**


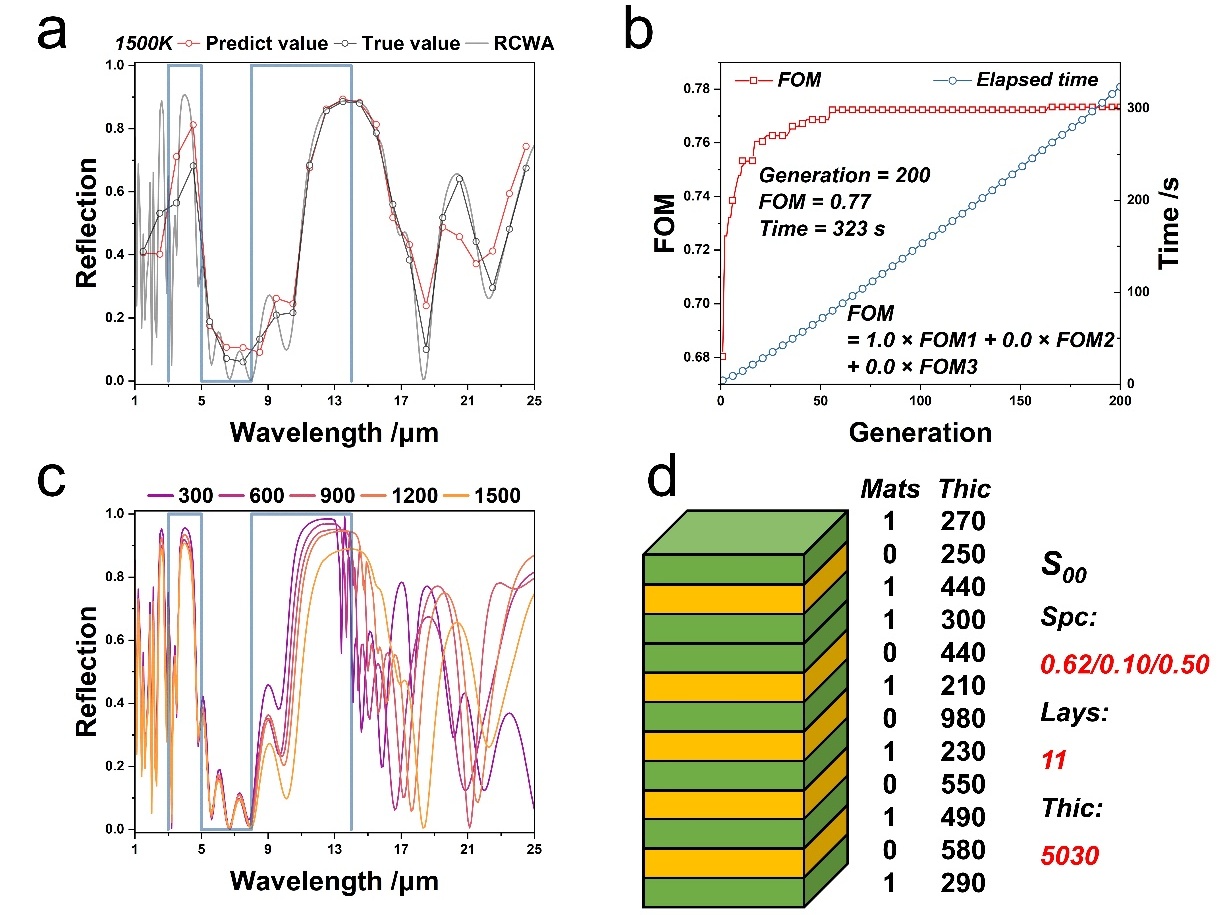


**Fig. S1** Detailed data for solution S_00_. **a**) Predicted values, true values, and RCWA value from the neural network at 1500 K; **b**) Evolution of FOM and elapsed time; **c**) Infrared spectrum across 300–1500 K; **d**) TPM layer architecture and spectral performance


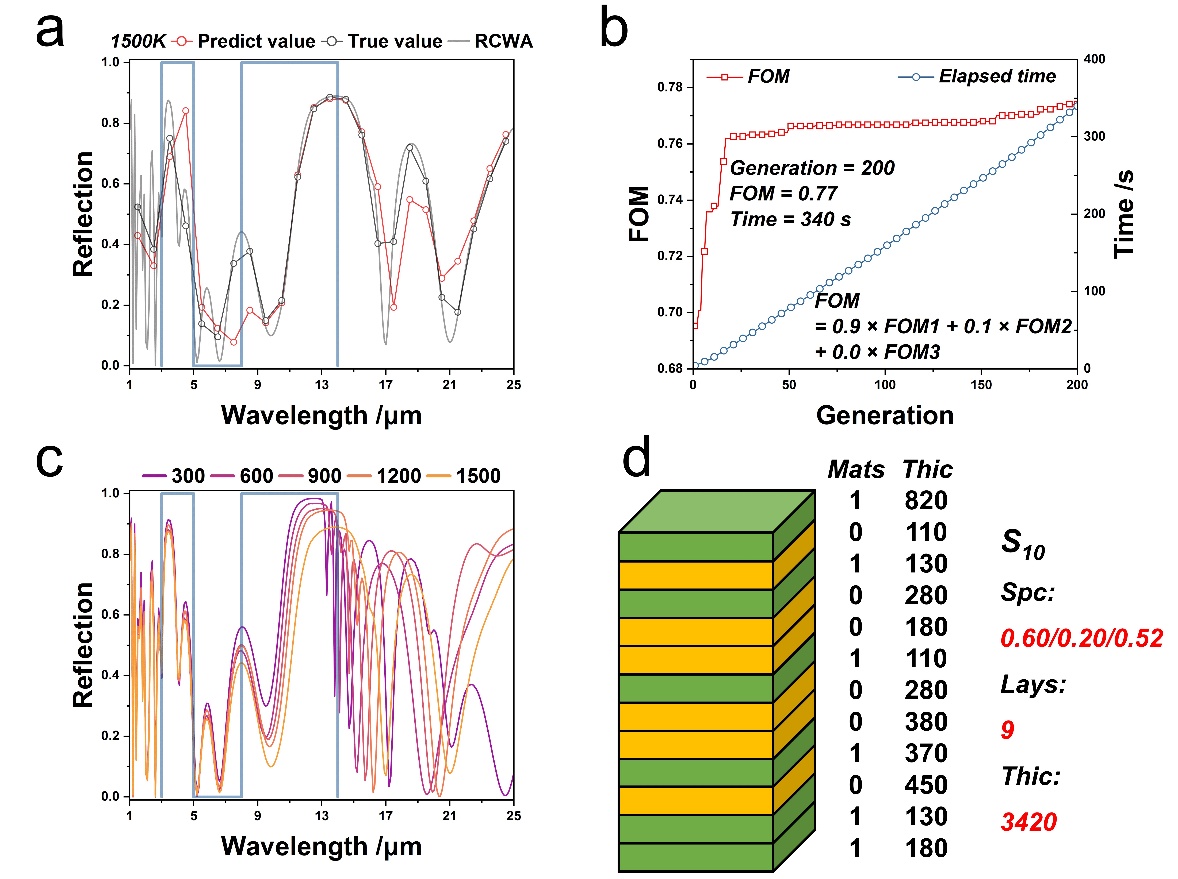


**Fig. S2** Detailed data for solution S_10_. **a**) Predicted values, true values, and RCWA value from the neural network at 1500 K; **b**) Evolution of FOM and elapsed time; **c**) Infrared spectrum across 300–1500 K; **d**) TPM layer architecture and spectral performance


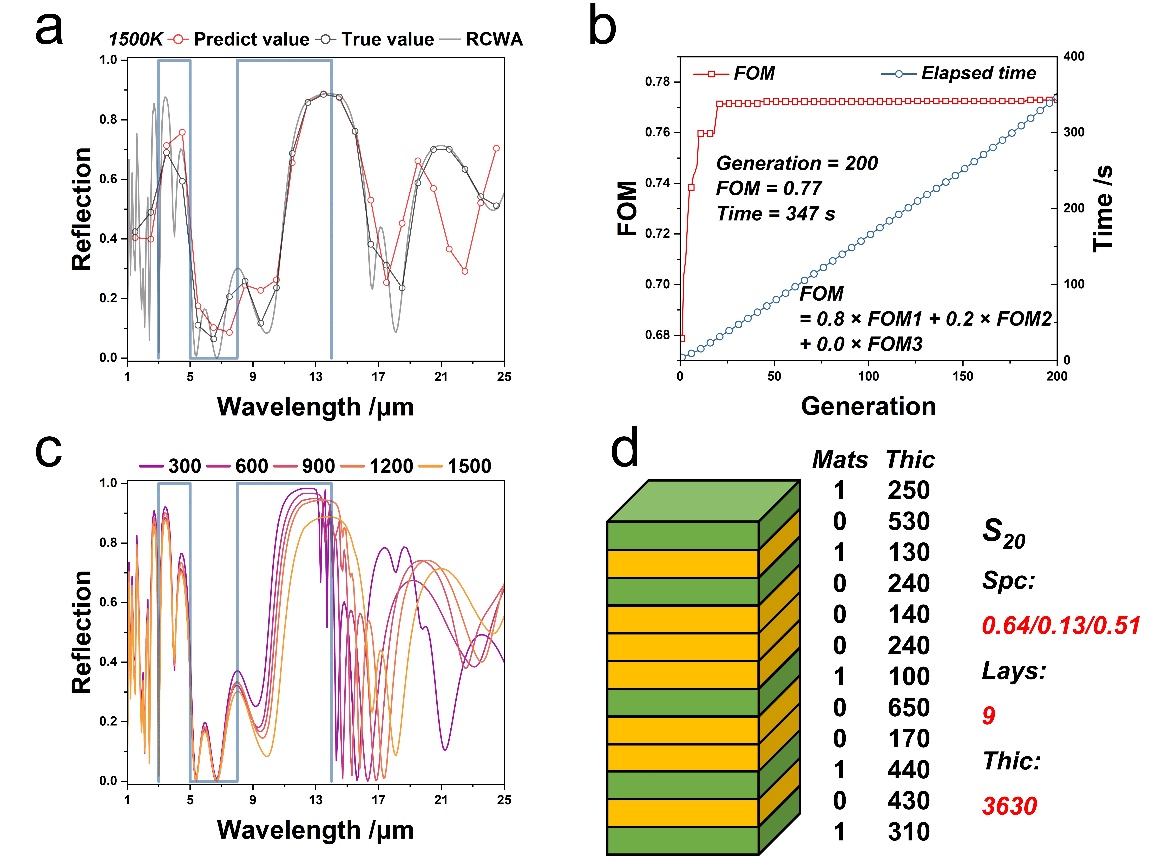


**Fig. S3** Detailed data for solution S_20_. **a**) Predicted values, true values, and RCWA value from the neural network at 1500 K; **b**) Evolution of FOM and elapsed time; **c**) Infrared spectrum across 300–1500 K; **d**) TPM layer architecture and spectral performance


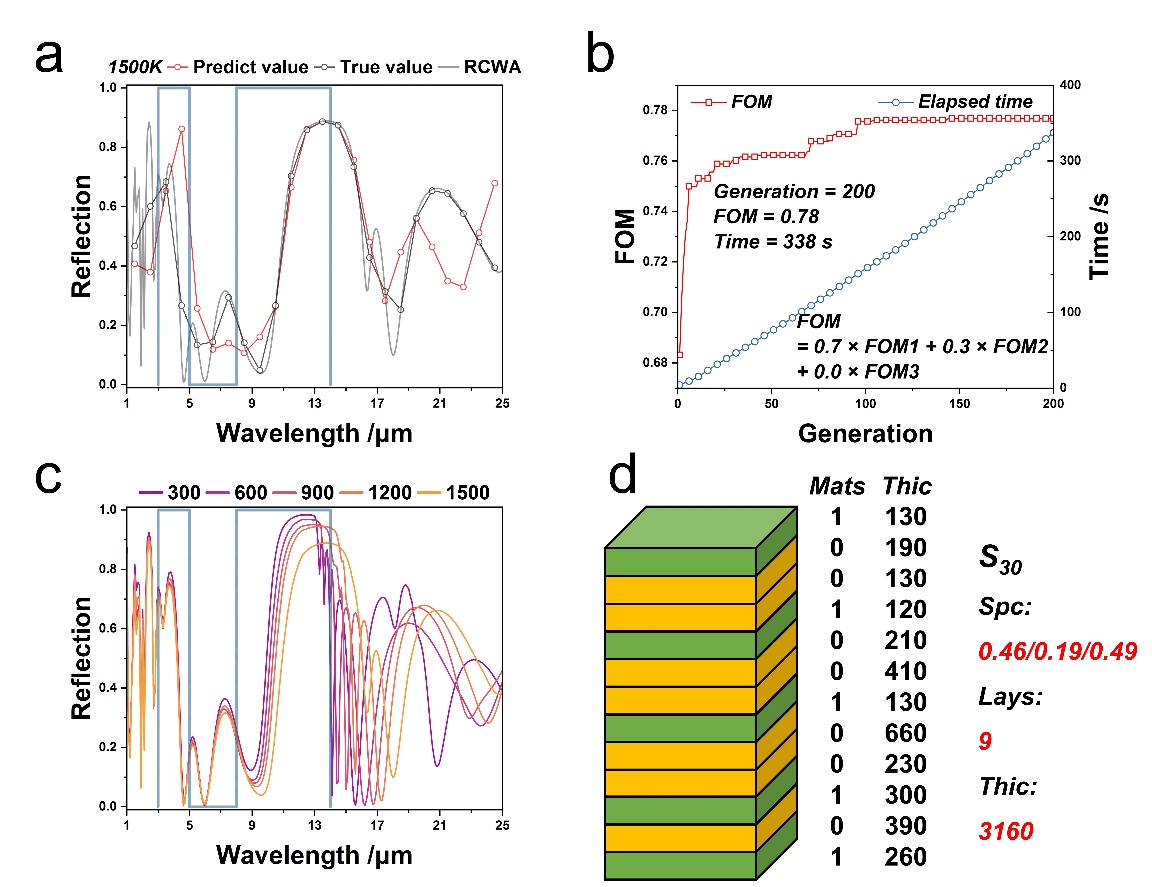


**Fig. S4** Detailed data for solution S_30_. **a**) Predicted values, true values, and RCWA value from the neural network at 1500 K; **b**) Evolution of FOM and elapsed time; **c**) Infrared spectrum across 300–1500 K; **d**) TPM layer architecture and spectral performance


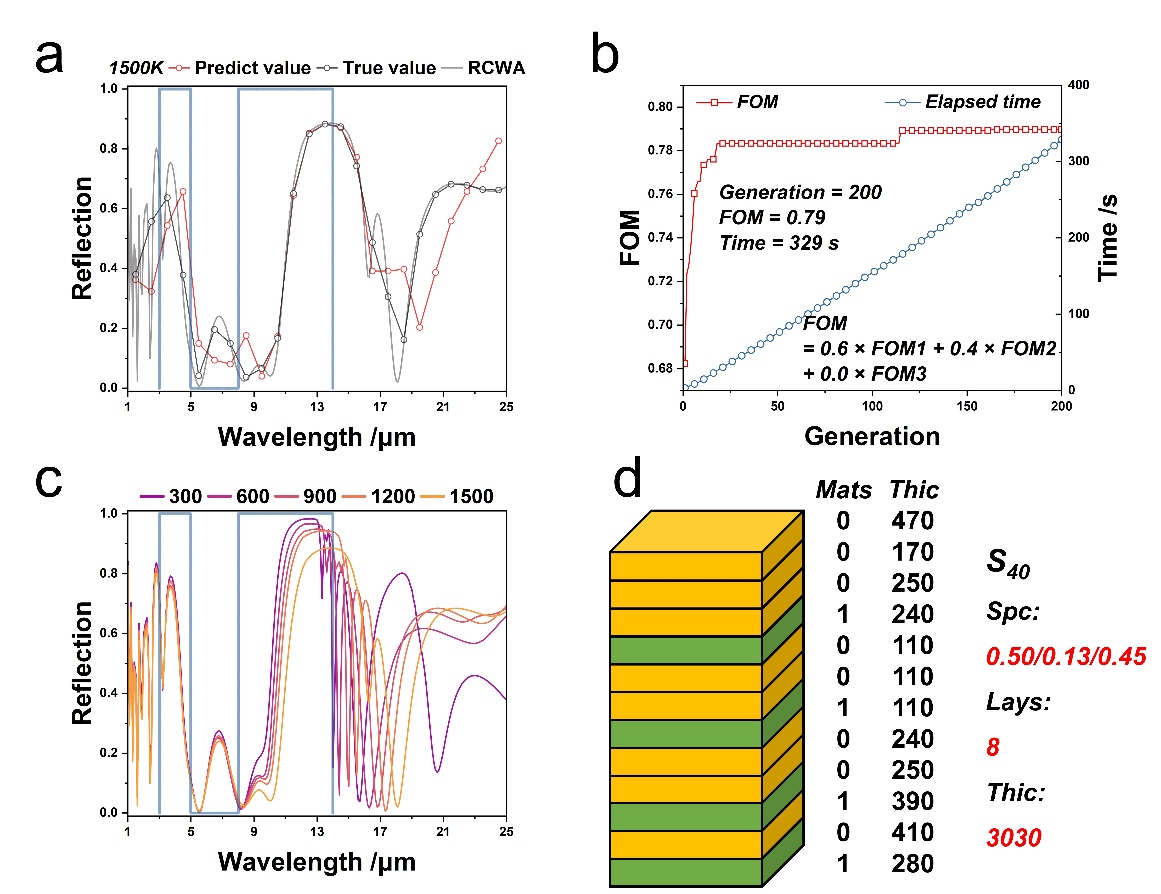


**Fig. S5** Detailed data for solution S_40_. **a**) Predicted values, true values, and RCWA value from the neural network at 1500 K; **b**) Evolution of FOM and elapsed time; **c**) Infrared spectrum across 300–1500 K; **d**) TPM layer architecture and spectral performance


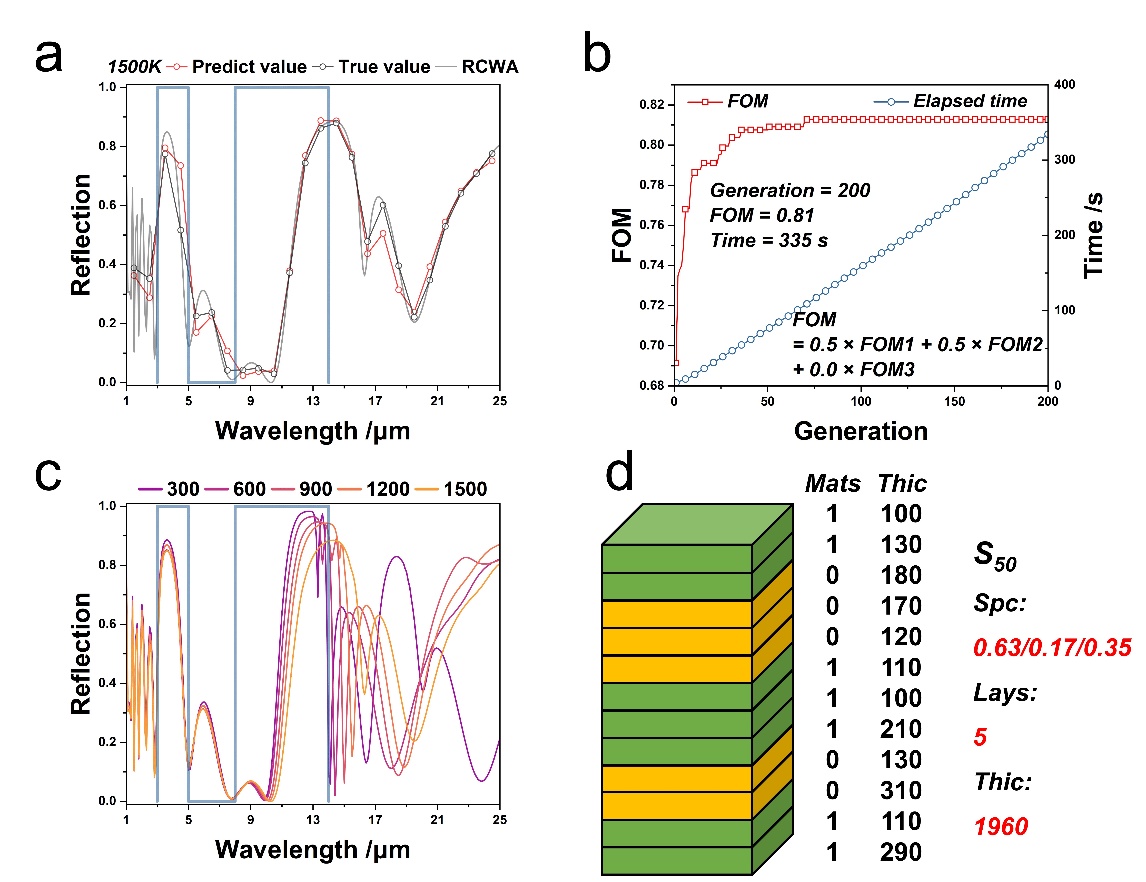


**Fig. S6** Detailed data for solution S_50_. **a**) Predicted values, true values, and RCWA value from the neural network at 1500 K; **b**) Evolution of FOM and elapsed time; **c**) Infrared spectrum across 300–1500 K; **d**) TPM layer architecture and spectral performance


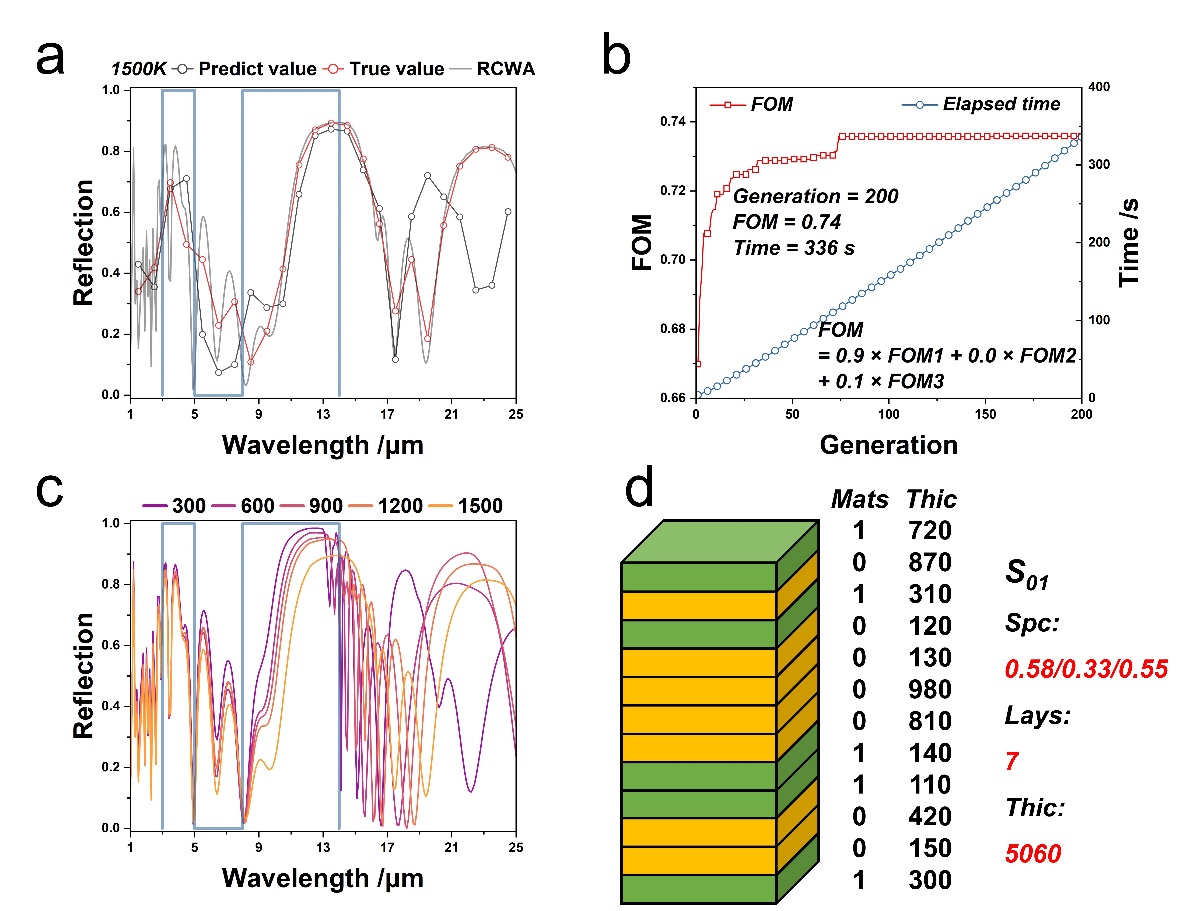


**Fig. S7** Detailed data for solution S_01_. **a**) Predicted values, true values, and RCWA value from the neural network at 1500 K; **b**) Evolution of FOM and elapsed time; **c**) Infrared spectrum across 300–1500 K; **d**) TPM layer architecture and spectral performance


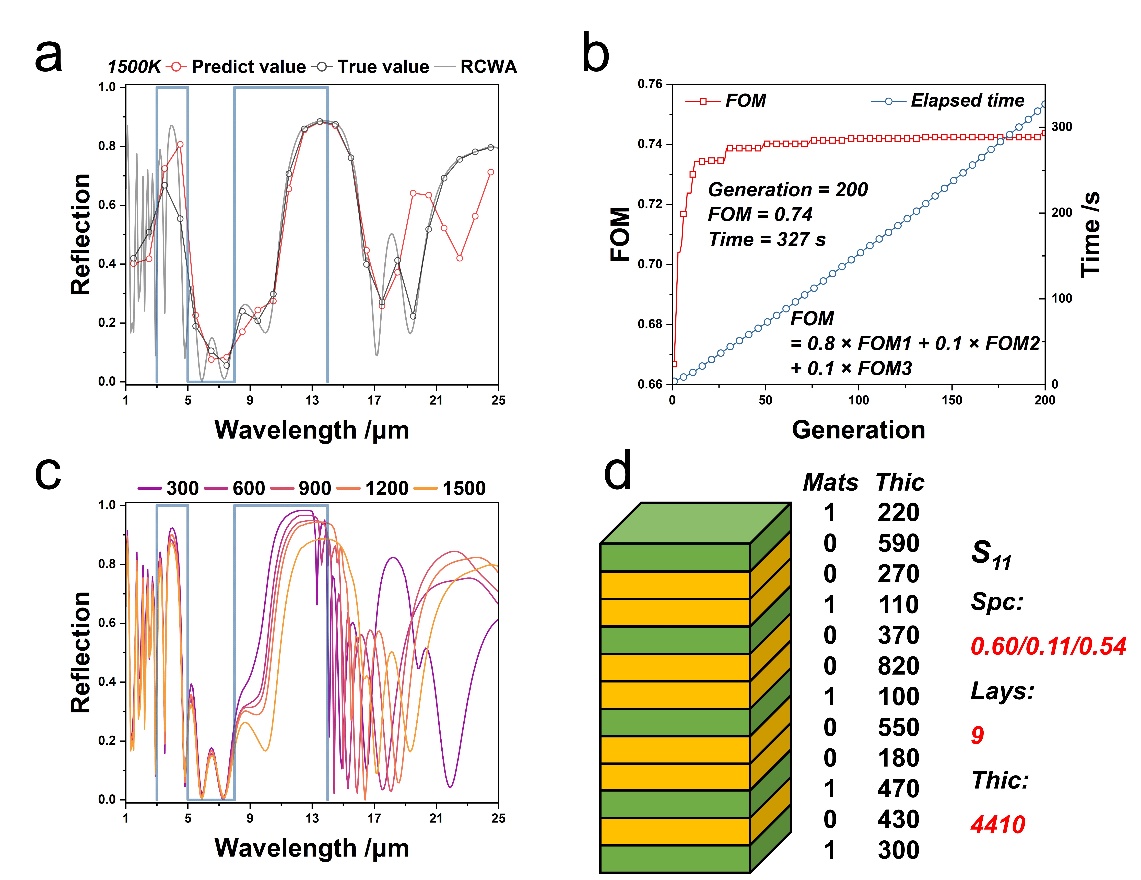


**Fig. S8** Detailed data for solution S_11_. **a**) Predicted values, true values, and RCWA value from the neural network at 1500 K; **b**) Evolution of FOM and elapsed time; **c**) Infrared spectrum across 300–1500 K; **d**) TPM layer architecture and spectral performance


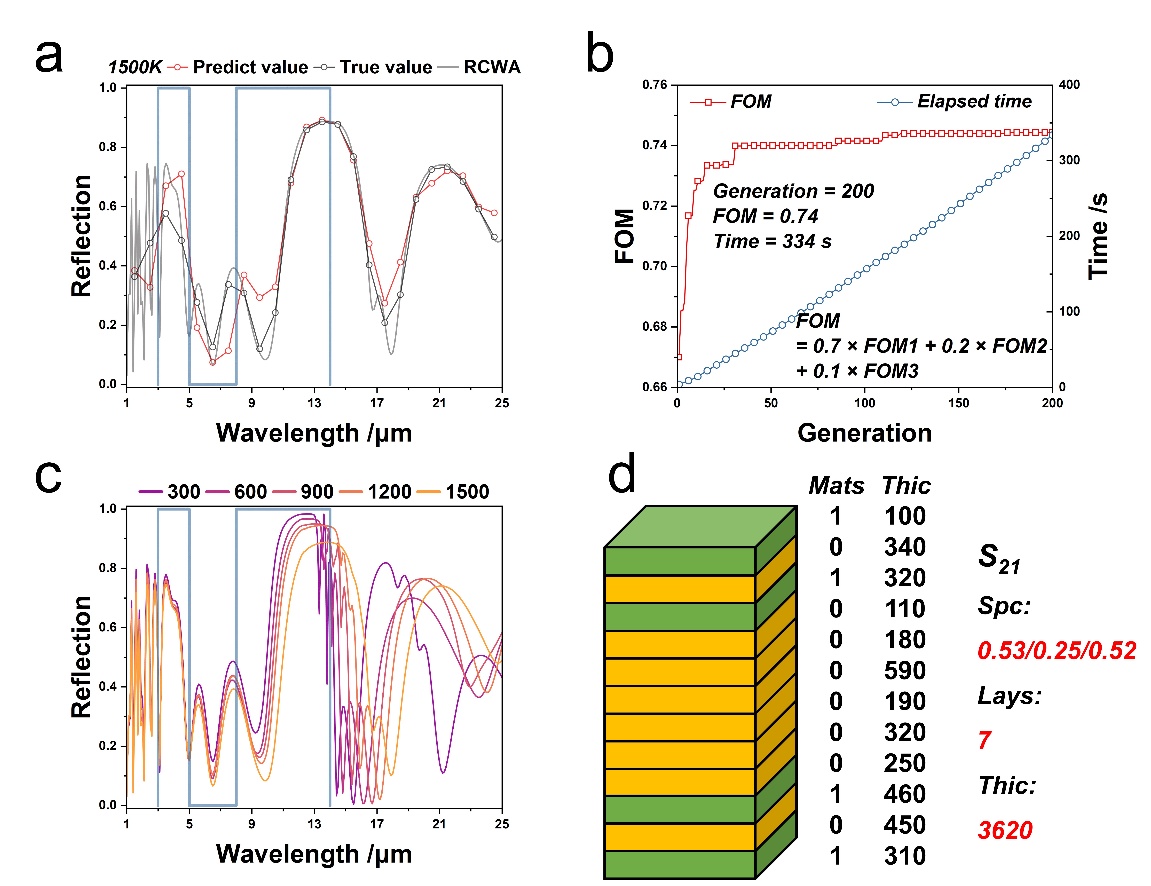


**Fig. S9** Detailed data for solution S_21_. **a**) Predicted values, true values, and RCWA value from the neural network at 1500 K; **b**) Evolution of FOM and elapsed time; **c**) Infrared spectrum across 300–1500 K; **d**) TPM layer architecture and spectral performance


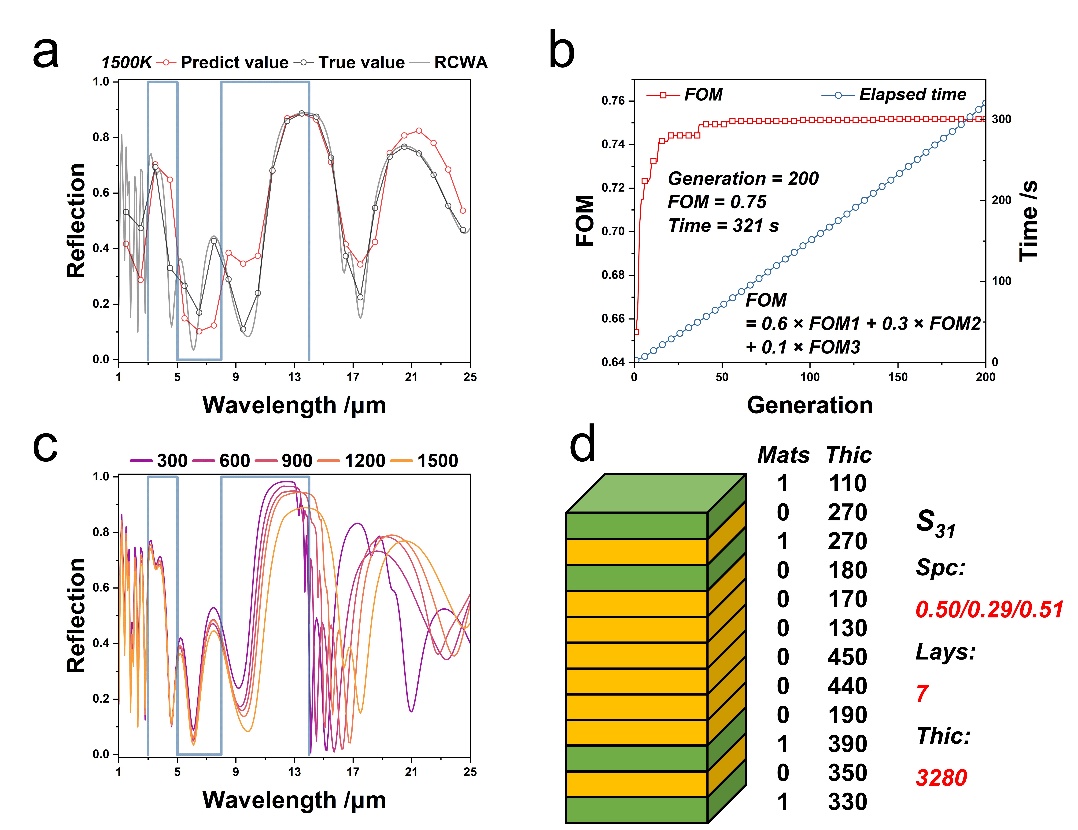


**Fig. S10** Detailed data for solution S_31_. **a**) Predicted values, true values, and RCWA value from the neural network at 1500 K; **b**) Evolution of FOM and elapsed time; **c**) Infrared spectrum across 300–1500 K; **d**) TPM layer architecture and spectral performance


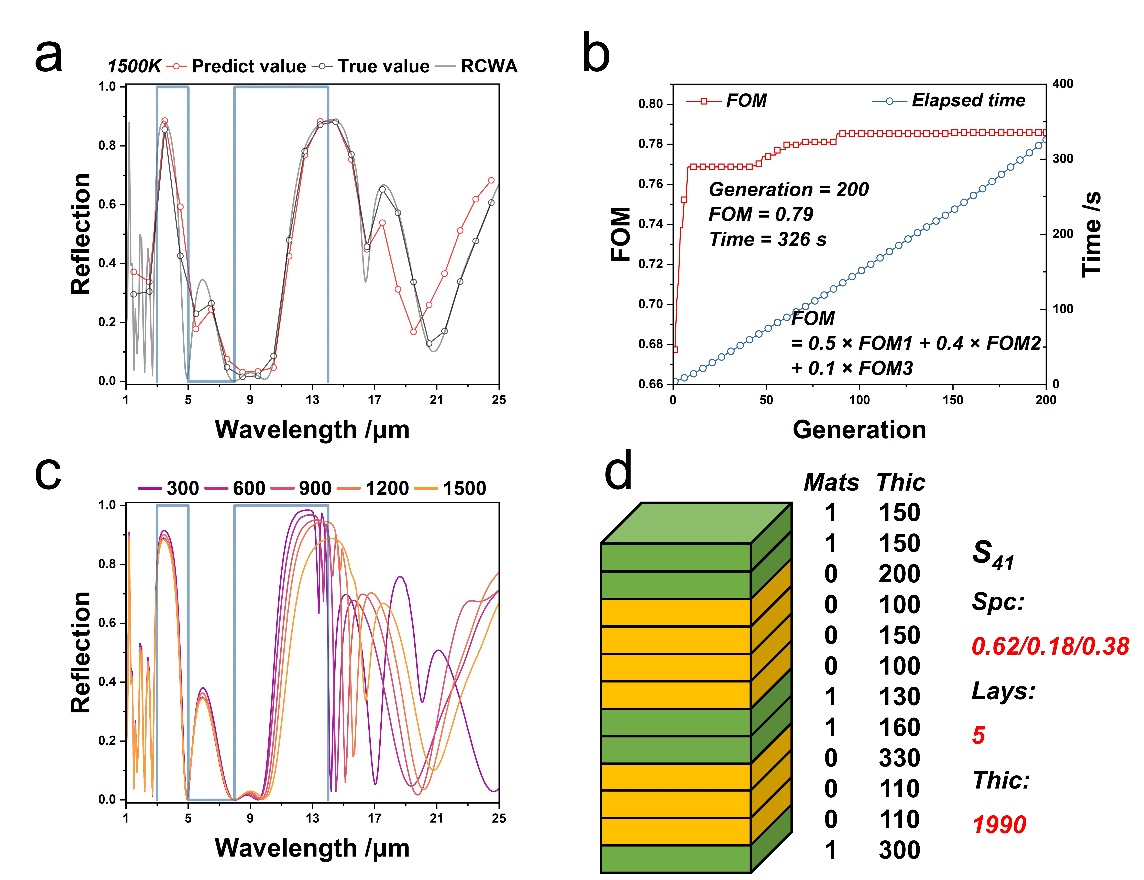


**Fig. S11** Detailed data for solution S_41_. **a**) Predicted values, true values, and RCWA value from the neural network at 1500 K; **b**) Evolution of FOM and elapsed time; **c**) Infrared spectrum across 300–1500 K; **d**) TPM layer architecture and spectral performance


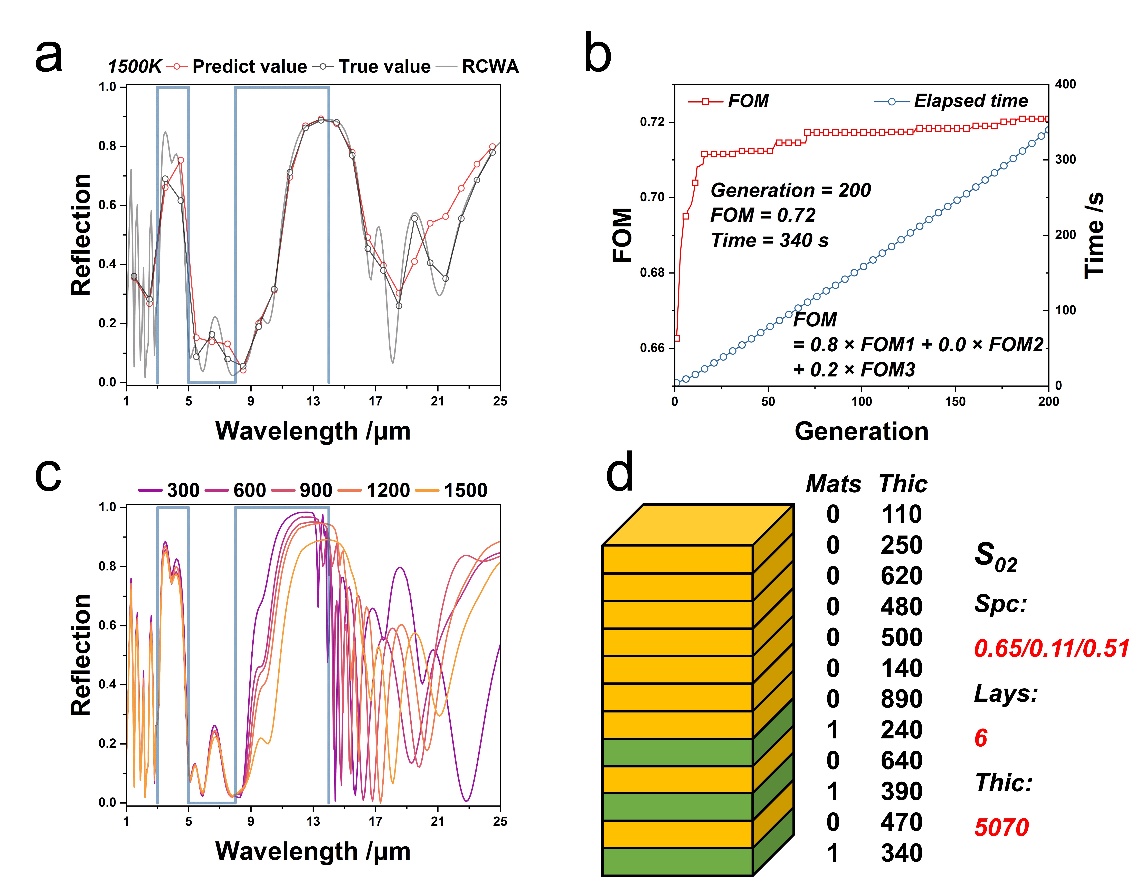


**Fig. S12** Detailed data for solution S_02_. **a**) Predicted values, true values, and RCWA value from the neural network at 1500 K; **b**) Evolution of FOM and elapsed time; **c**) Infrared spectrum across 300–1500 K; **d**) TPM layer architecture and spectral performance


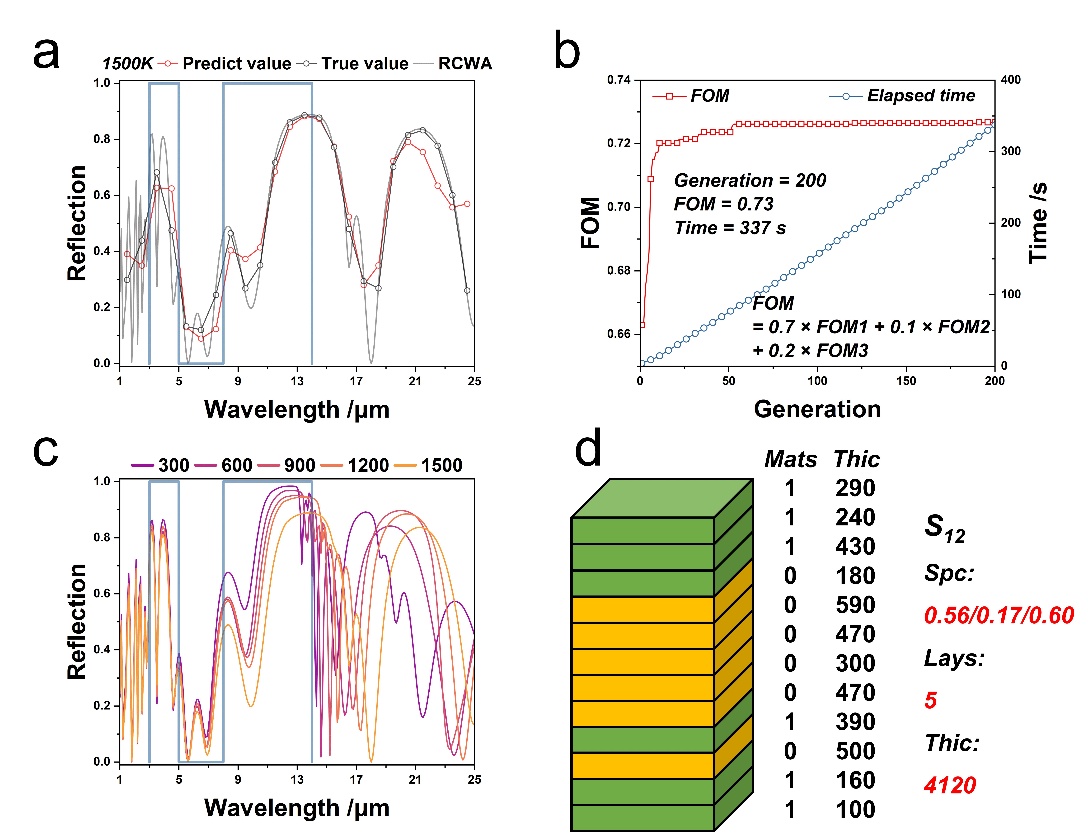


**Fig. S13** Detailed data for solution S_12_. **a**) Predicted values, true values, and RCWA value from the neural network at 1500 K; **b**) Evolution of FOM and elapsed time; **c**) Infrared spectrum across 300–1500 K; **d**) TPM layer architecture and spectral performance


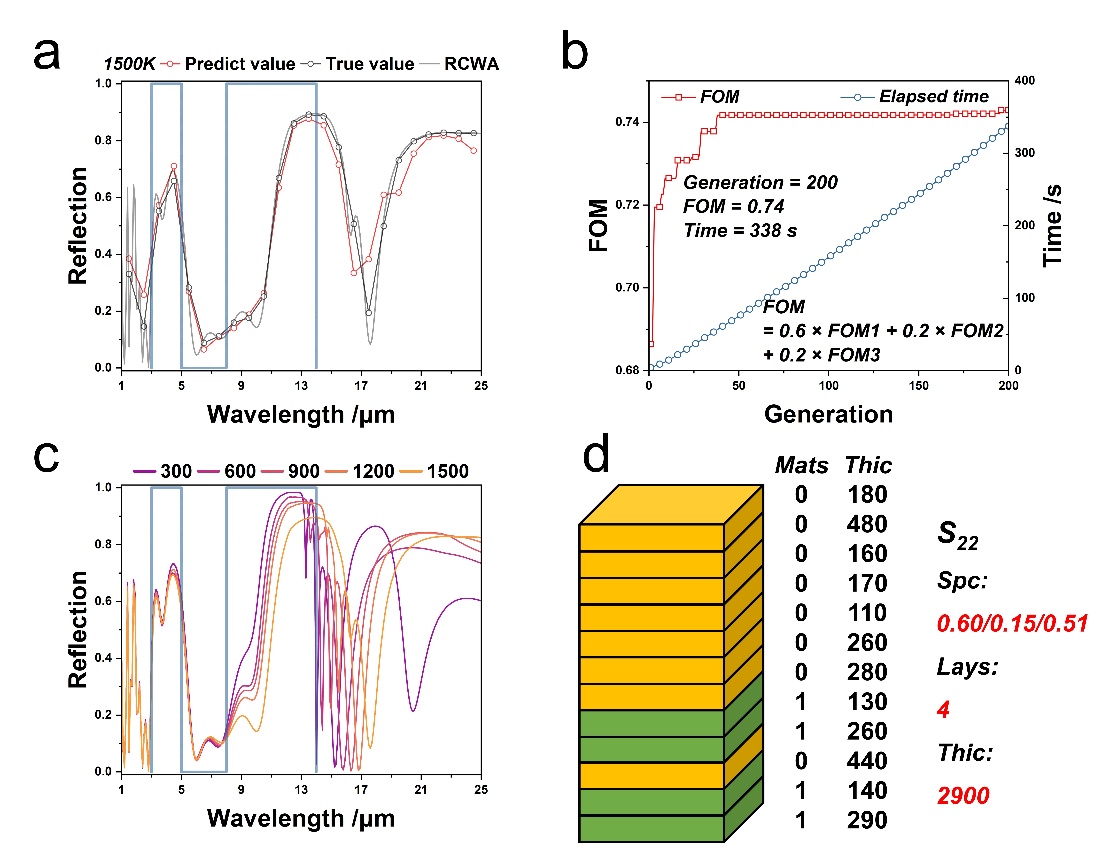


**Fig. S14** Detailed data for solution S_22_. **a**) Predicted values, true values, and RCWA value from the neural network at 1500 K; **b**) Evolution of FOM and elapsed time; **c**) Infrared spectrum across 300–1500 K; **d**) TPM layer architecture and spectral performance


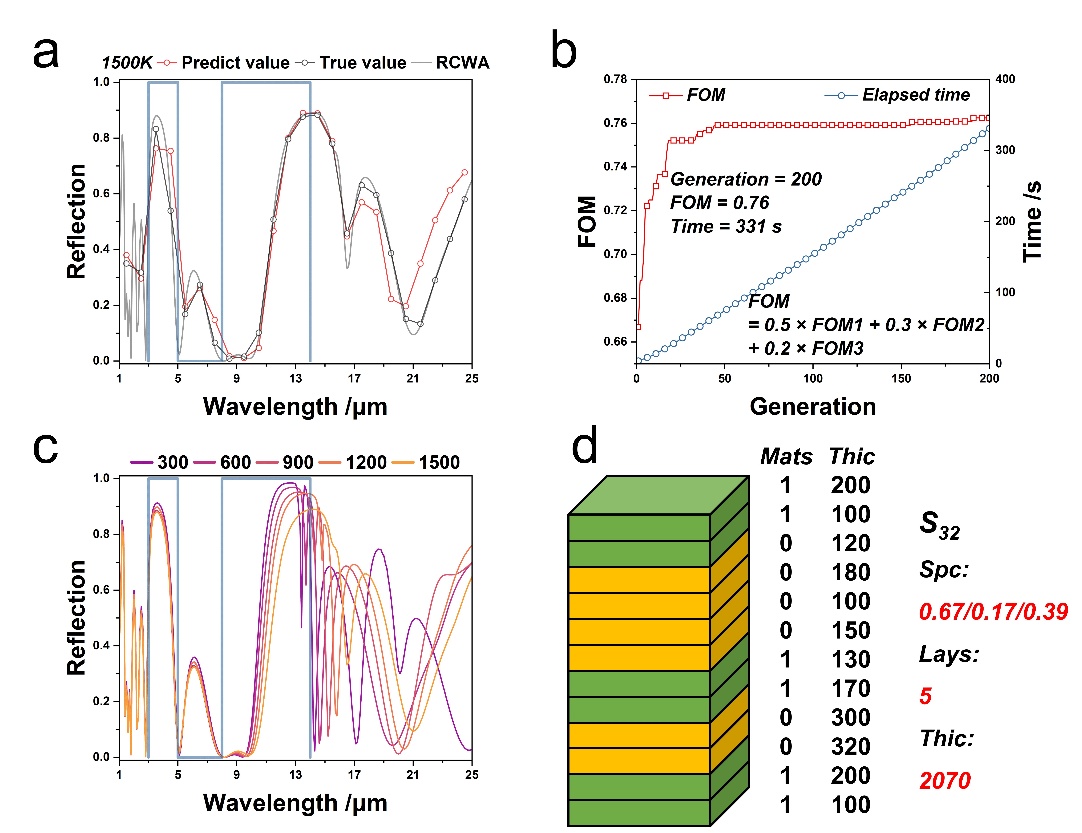


**Fig. S15** Detailed data for solution S_32_. **a**) Predicted values, true values, and RCWA value from the neural network at 1500 K; **b**) Evolution of FOM and elapsed time; **c**) Infrared spectrum across 300–1500 K; **d**) TPM layer architecture and spectral performance


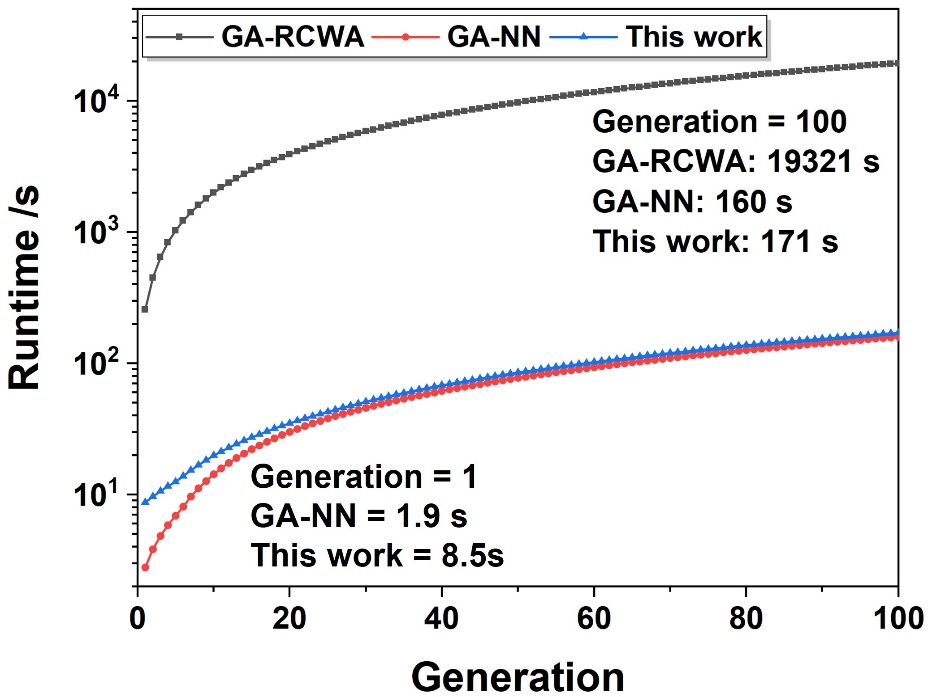


**Fig. S16** Runtime of the GA‑RCWA, GA‑NN, and multi‑objective algorithms


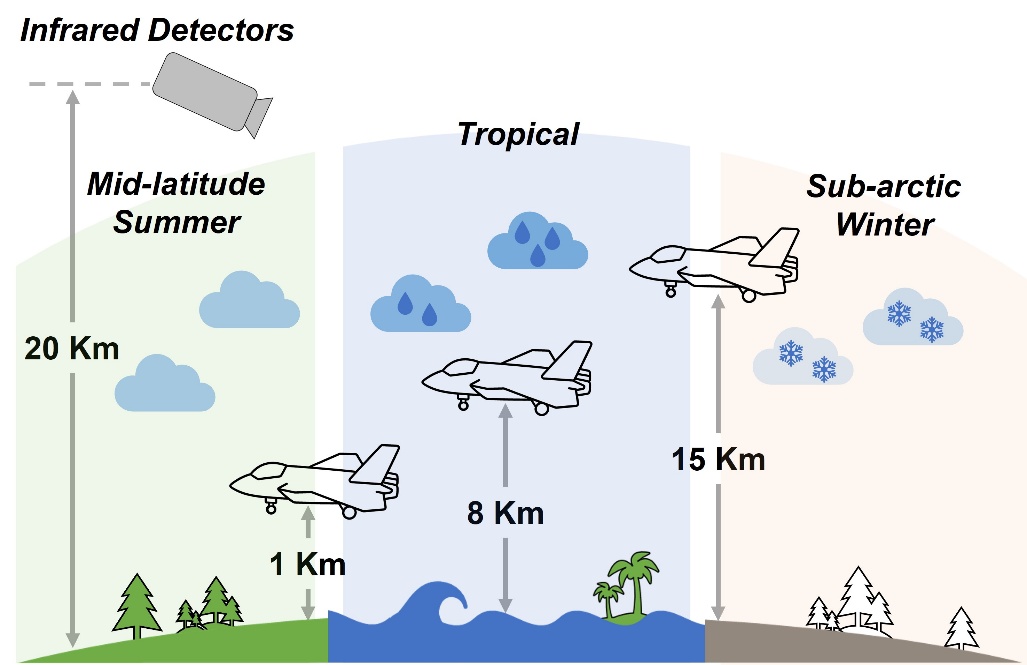


**Fig. S17** Schematic of the atmospheric transmission model


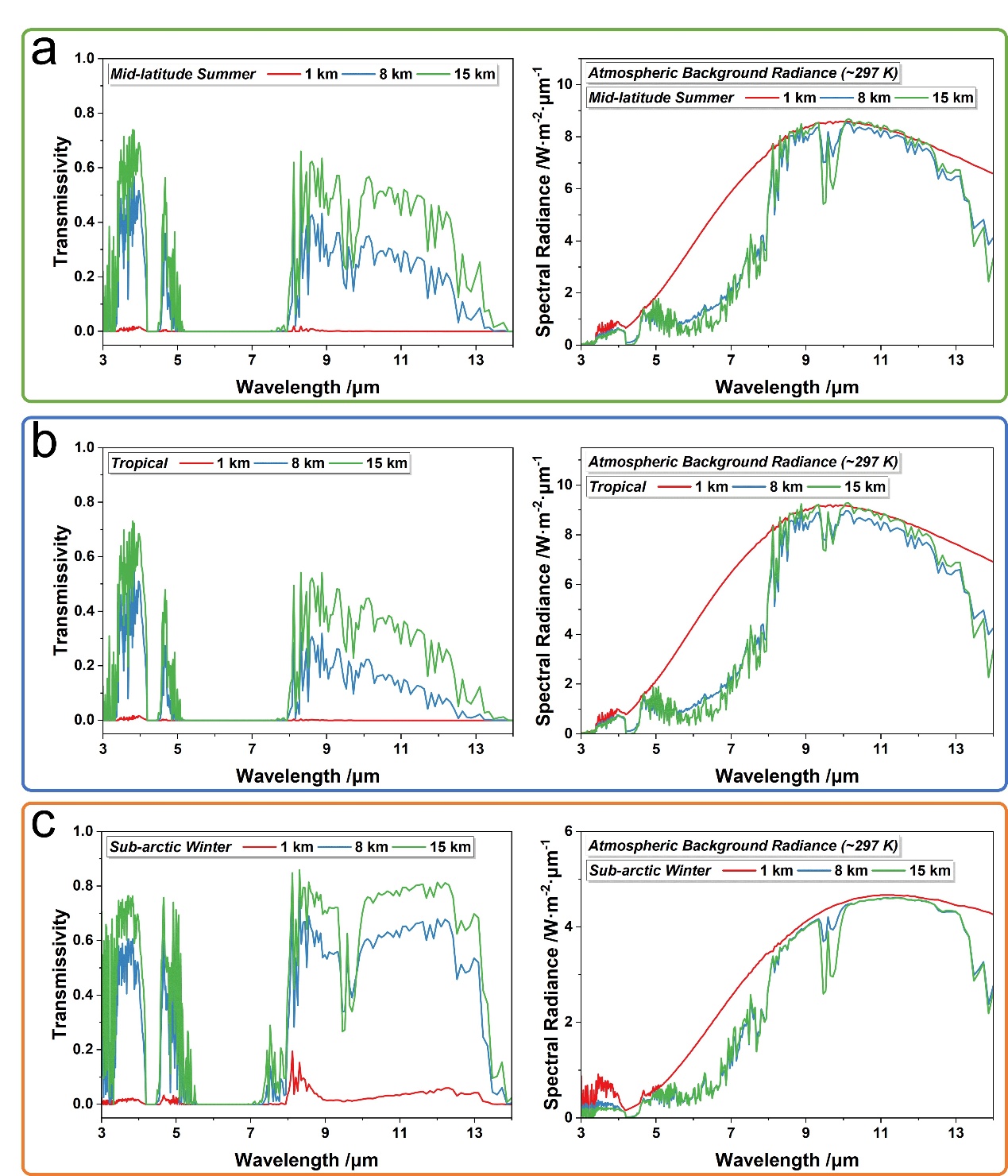


**Fig. S18** Atmospheric transmittance and background radiance


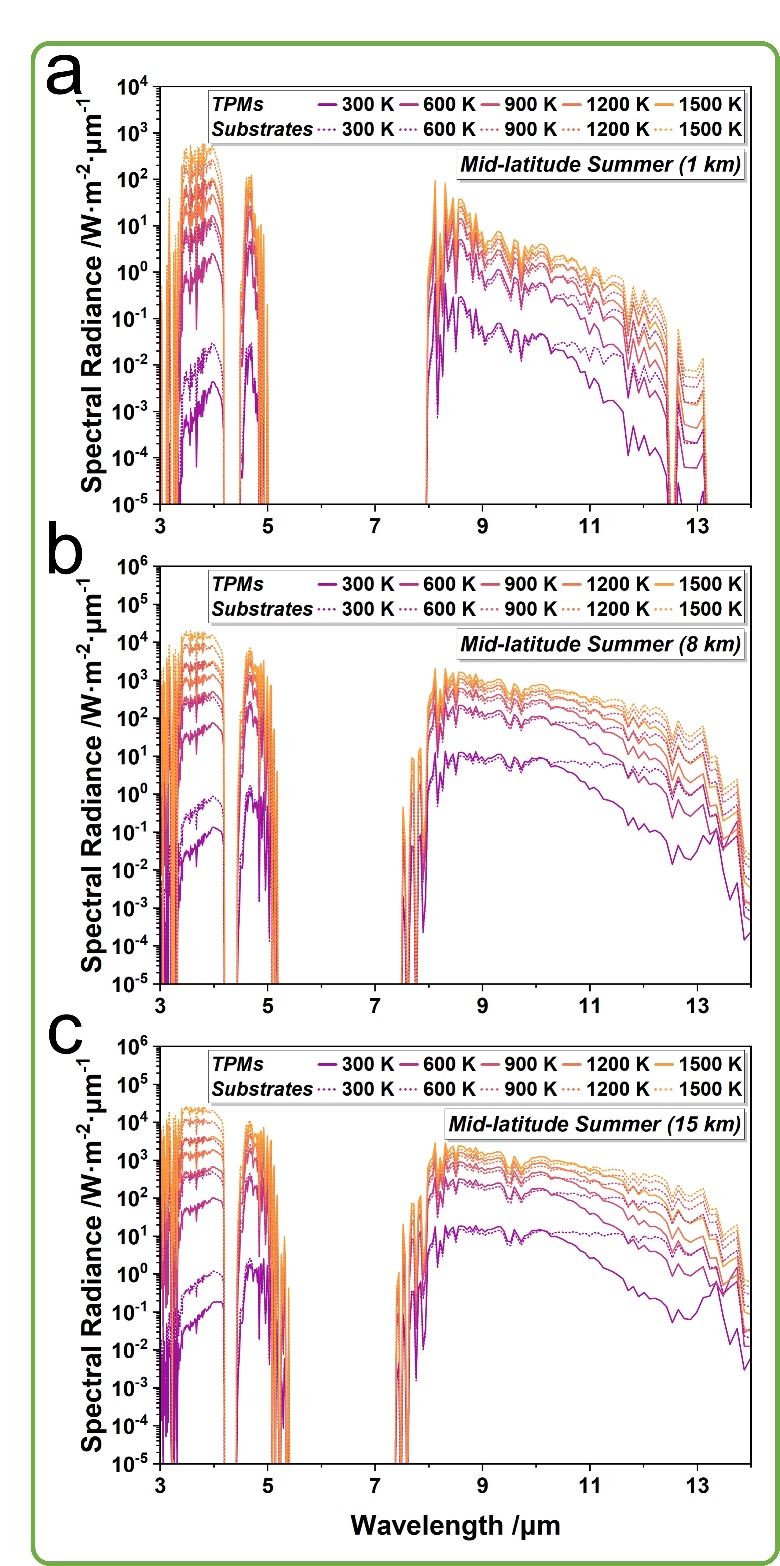


**Fig. S19** Spectral radiance of sample S32 vs. metal oxide substrates under the mid-latitude summer model


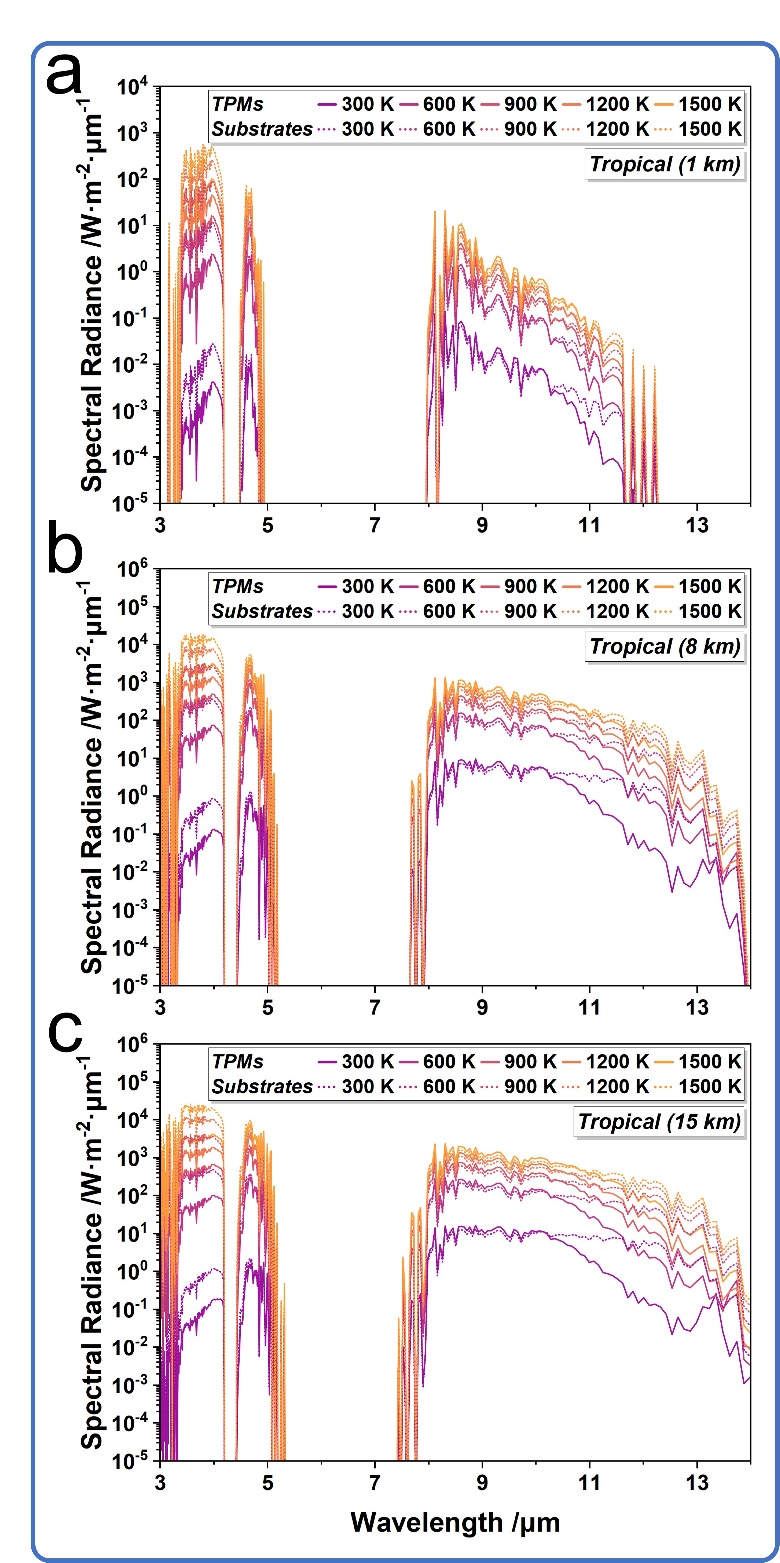


**Fig. S20** Spectral radiance of sample S32 vs. metal oxide substrates under the tropical model


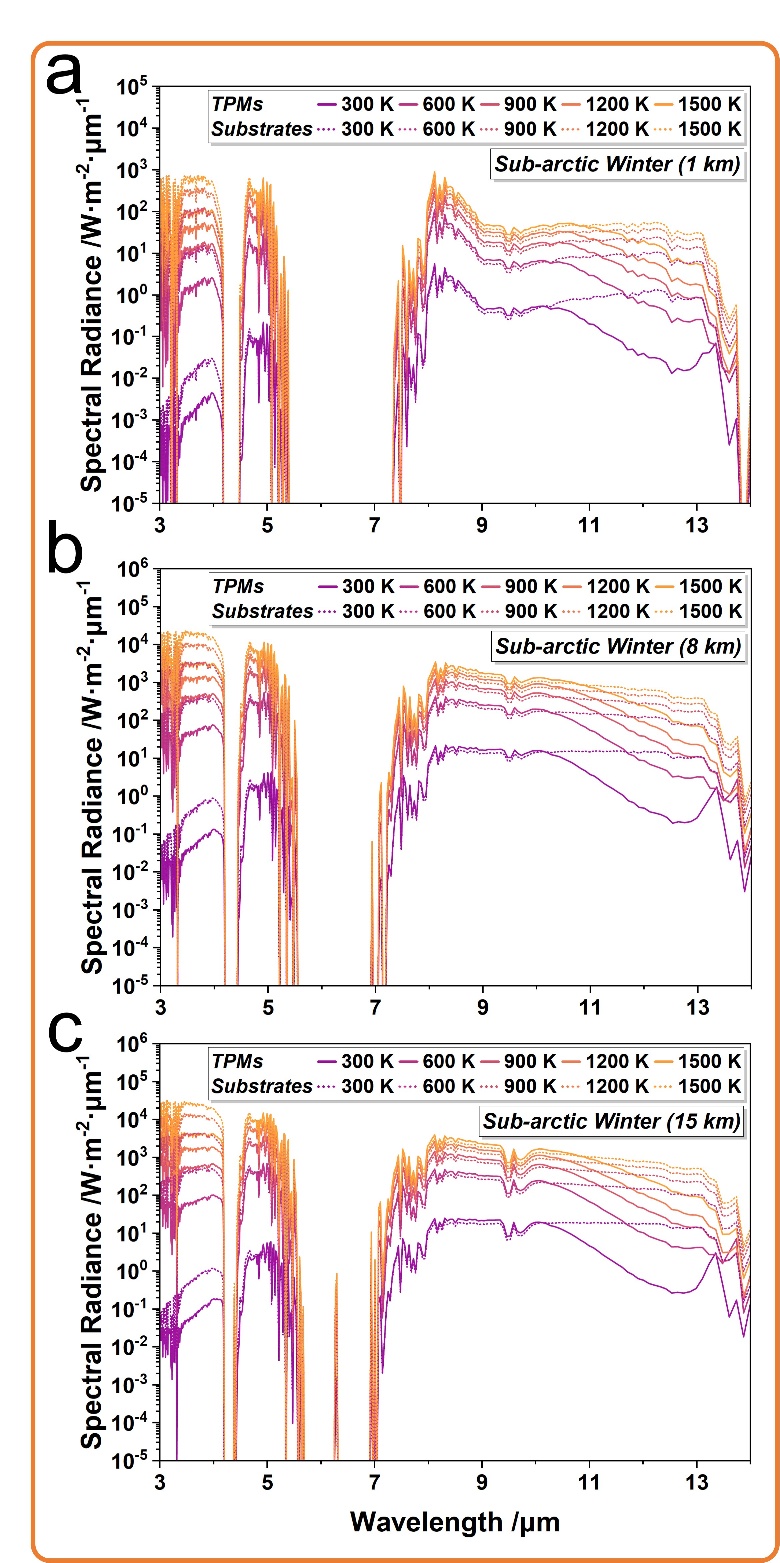


**Fig. S21** Spectral radiance of sample S32 vs. metal oxide substrates under the sub-arctic winter model


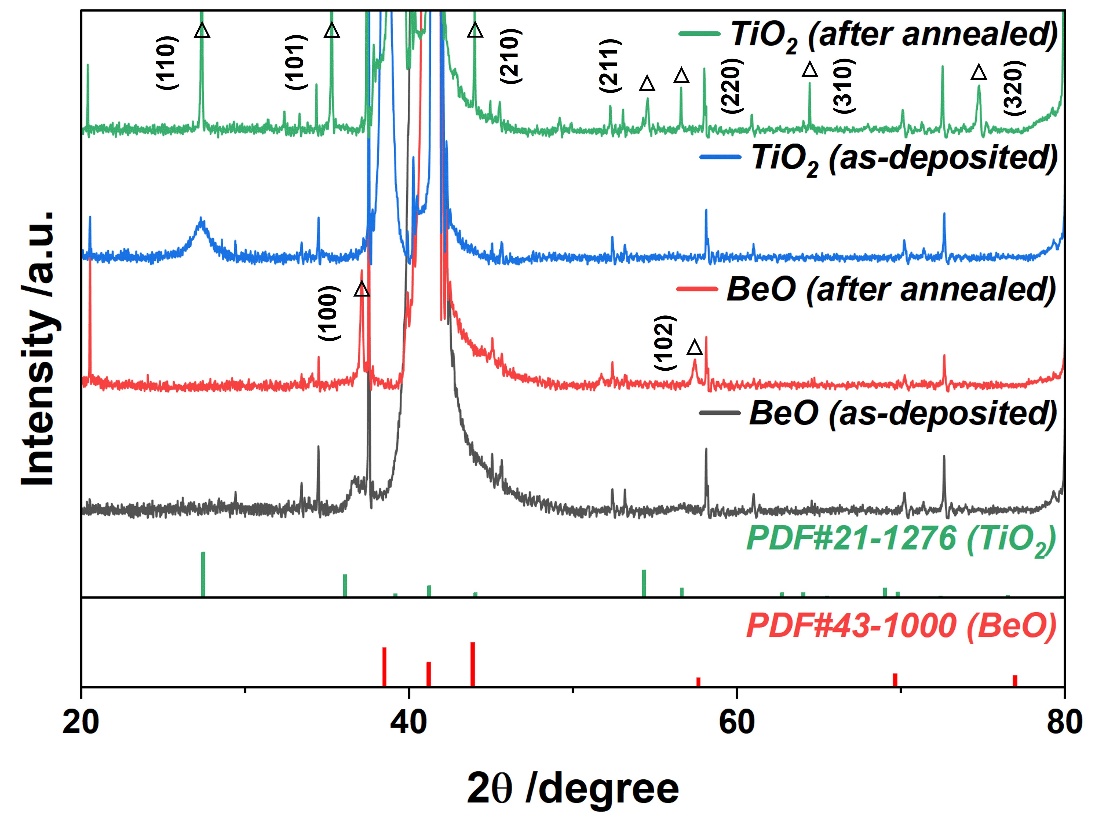


**Fig. S22** X-ray diffraction patterns of BeO and TiO_2_ before and after annealing


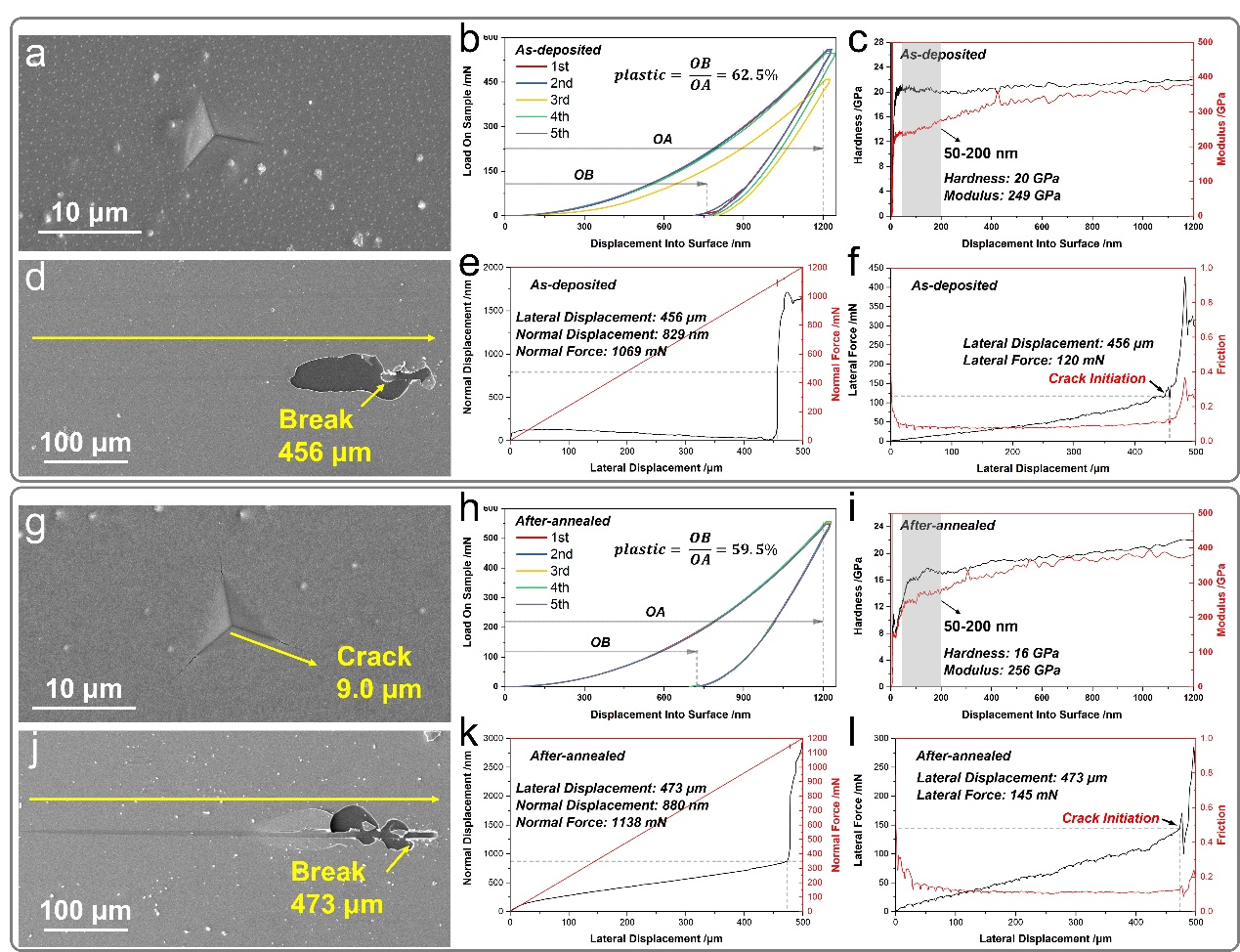


**Fig. S23** Mechanical properties of 2-μm YSZ. **a-c**) Nano-indentation testing before annealing; **d-f**) Nano-scratch testing before annealing; **g-i**) Nano-indentation after annealing; **j-l**) Nano-scratch testing after annealing

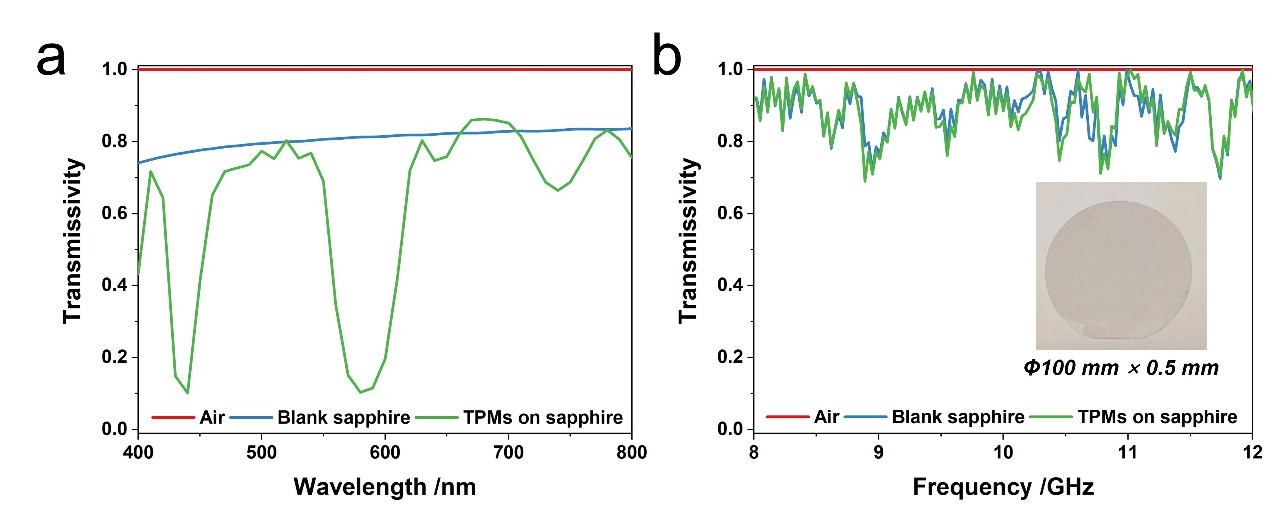


**Fig. S24** Comparative transmittance spectra of air, blank sapphire, and TPMs on sapphire in **a**) visible and **b**) microwave bands


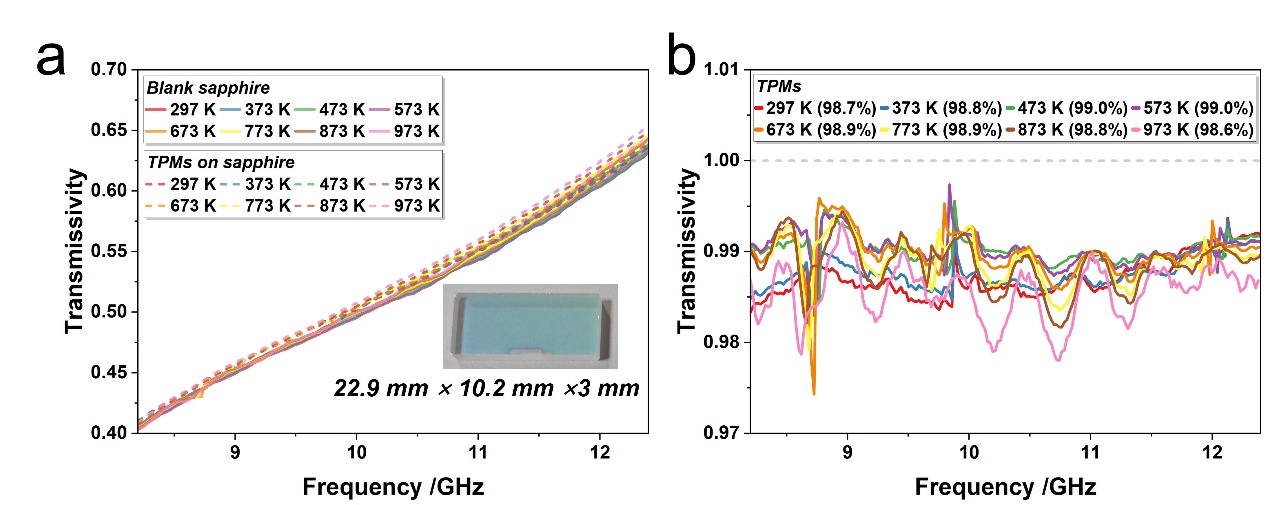


**Fig. S25** Comparative transmittance spectra of blank sapphire and TPMs on sapphire in microwave bands across 300 to 1000 K


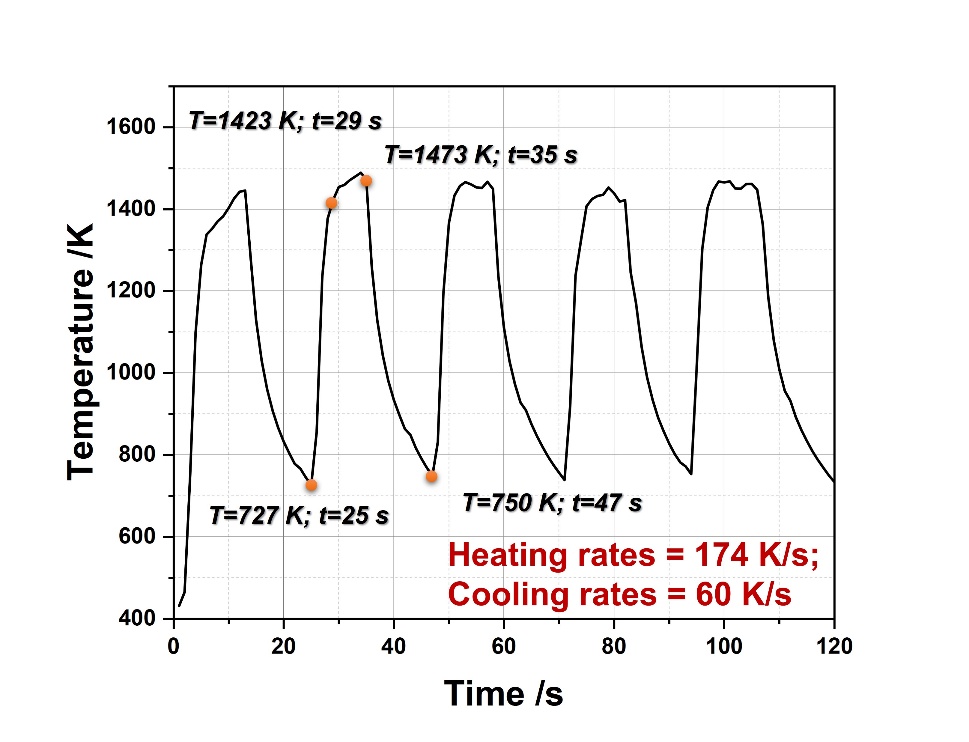


**Fig. S26** Temperature-time curve of the thermal shock test


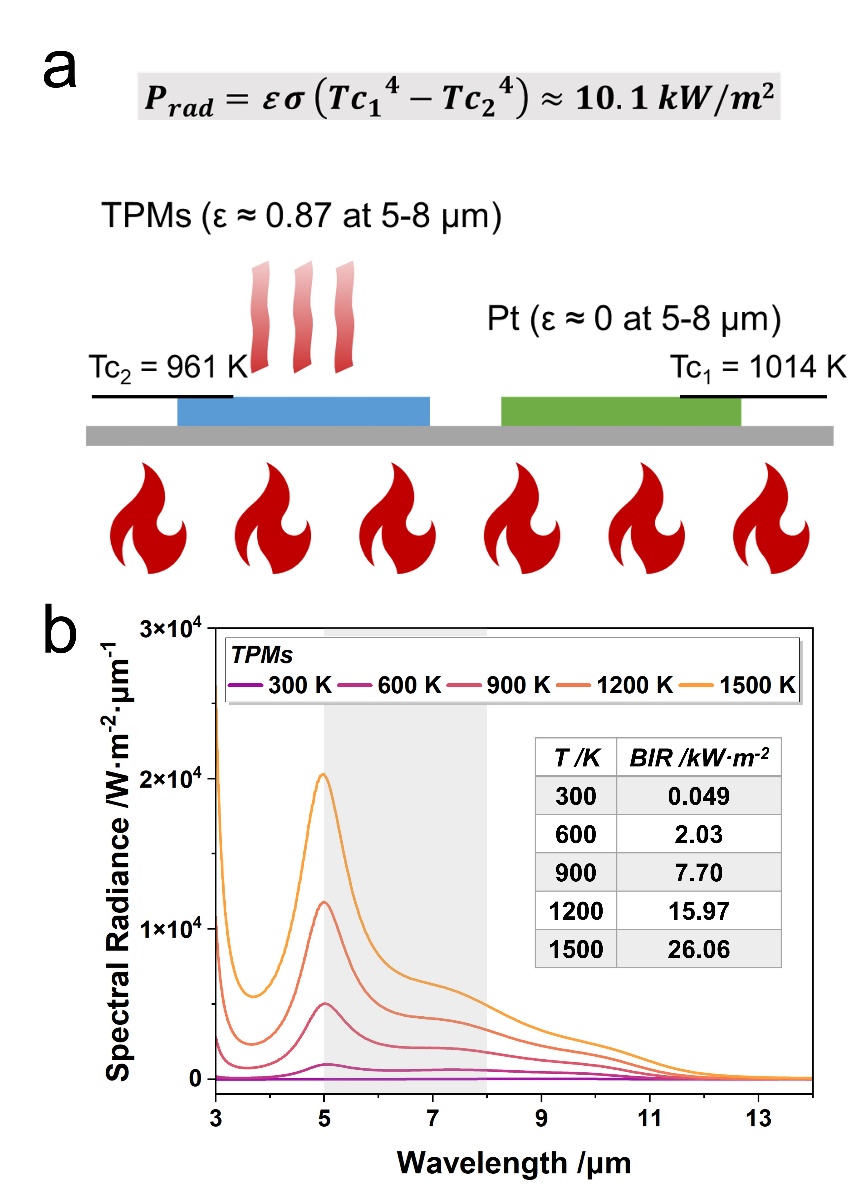


**Fig. S27** Radiative cooling performance of the TPMs. a) Measured radiative cooling power (P_rad_) within the 5-8 μm spectral band at 1014 K. b) Theoretical radiative cooling power (BIR, band-integrated radiance) within the 5-8 μm band across the 300-1500 K temperature range


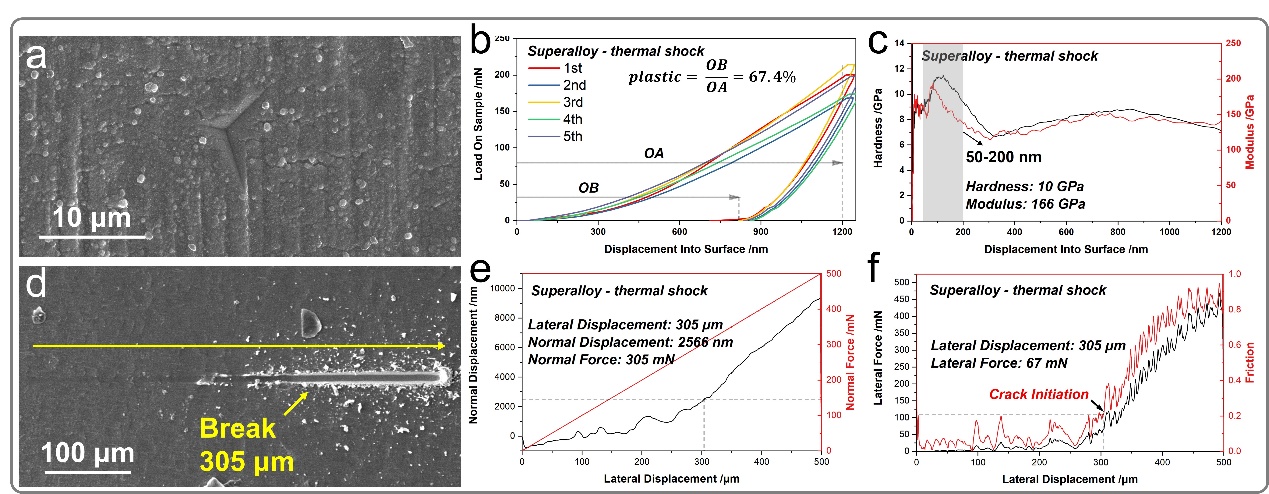


**Fig. S28** Mechanical properties of TPM on superalloy substrate. a-c) Nano-indentation and d-f) Nano-scratch testing after thermal shock testing

**Table S1** Material‑property trade‑offs for high‑temperature dielectric layers

| Materials | *n* (mid‑IR) | Melting point  /K | CTE  /×10^-6^· K^-1^ | Stability | Reflectivity  (8-14 μm, 300 K) |
| --- | --- | --- | --- | --- | --- |
| BeO | ~1.7-1.9 | ~2,800 | ~9 | Excellent | ~0.7 |
| TiO_2_ | ~2.4-2.6 | ~2,130 | ~9 | Excellent | ~0.1 |
| Al_2_O_3_ | ~1.7-1.8 | ~2,323 | ~8 | Excellent | ~0.3 |
| MgF_2_ | ~1.3-1.4 | ~1,536 | ~13-15 | Good | ~0 |
| SrTiO_3_ | ~2.3-2.5 | ~2,350 | ~10-12 | Good | ~0.2 |

**Table S2** RMSE between the GA-NN and RCWA methods

| Solution | 300 K | 600 K | 900 K | 1200 K | 1500 K |
| --- | --- | --- | --- | --- | --- |
| S_00_ | 0.113 | 0.066 | 0.083 | 0.082 | 0.066 |
| S_10_ | **0.147** | **0.178** | **0.18** | **0.186** | **0.153** |
| S_20_ | 0.115 | 0.087 | 0.088 | 0.092 | 0.075 |
| S_30_ | **0.176** | **0.13** | **0.135** | **0.131** | **0.193** |
| S_40_ | 0.132 | 0.095 | 0.094 | 0.077 | 0.189 |
| S_50_ | **0.061** | **0.042** | **0.081** | **0.078** | **0.073** |
| S_01_ | **0.181** | **0.167** | **0.177** | **0.177** | **0.152** |
| S_11_ | 0.104 | 0.079 | 0.092 | 0.088 | 0.085 |
| S_21_ | **0.164** | **0.147** | **0.142** | **0.147** | **0.121** |
| S_31_ | 0.129 | 0.116 | 0.11 | 0.113 | 0.164 |
| S_41_ | **0.078** | **0.032** | **0.037** | **0.064** | **0.059** |
| S_02_ | 0.089 | 0.062 | 0.059 | 0.052 | 0.05 |
| S_12_ | 0.12 | 0.08 | 0.082 | 0.084 | 0.075 |
| S_22_ | **0.133** | **0.041** | **0.029** | **0.033** | **0.023** |
| S_32_ | **0.036** | **0.044** | **0.046** | **0.041** | **0.076** |

**Table S3** RMSE between the temperature-dependent infrared engineering model and the baseline model

| Solution | 300 K | 600 K | 900 K | 1200 K | 1500 K |
| --- | --- | --- | --- | --- | --- |
| S_00_ | 0 | 0.088 | 0.104 | 0.134 | **0.217** |
| S_10_ | 0 | 0.093 | 0.11 | 0.137 | **0.201** |
| S_20_ | 0 | 0.081 | 0.105 | 0.135 | **0.212** |
| S_30_ | 0 | 0.06 | 0.097 | 0.131 | **0.204** |
| S_40_ | 0 | 0.064 | 0.099 | 0.134 | **0.21** |
| S_50_ | 0 | 0.066 | 0.097 | 0.13 | **0.187** |
| S_01_ | 0 | 0.093 | 0.103 | 0.131 | **0.231** |
| S_11_ | 0 | 0.083 | 0.1 | 0.128 | **0.219** |
| S_21_ | 0 | 0.087 | 0.11 | 0.139 | **0.214** |
| S_31_ | 0 | 0.073 | 0.101 | 0.131 | **0.206** |
| S_41_ | 0 | 0.065 | 0.092 | 0.126 | **0.185** |
| S_02_ | 0 | 0.098 | 0.116 | 0.145 | **0.239** |
| S_12_ | 0 | 0.083 | 0.096 | 0.123 | **0.215** |
| S_22_ | 0 | 0.077 | 0.1 | 0.13 | **0.211** |
| S_32_ | 0 | 0.074 | 0.097 | 0.129 | **0.187** |

**Table S4** The solution set of calculation results

| Solution | FOM_1_ | FOM_2_ | FOM_3_ | FOM |
| --- | --- | --- | --- | --- |
| S_00_ | **0.726149** | 0.724382 | 0.39985 | **0.726149** |
| S_10_ | 0.700473 | 0.803136 | 0.472367 | 0.677662 |
| S_20_ | **0.728331** | 0.792397 | 0.472367 | 0.677138 |
| S_30_ | 0.65116 | 0.816634 | 0.472367 | 0.597522 |
| S_40_ | 0.670991 | **0.823468** | 0.513417 | 0.607961 |
| S_50_ | 0.693503 | **0.881931** | **0.659241** | 0.676372 |
| S_01_ | 0.670991 | 0.722991 | 0.558035 | 0.676191 |
| S_11_ | **0.722527** | 0.753752 | 0.472367 | 0.700634 |
| S_21_ | 0.66631 | 0.792905 | 0.558035 | 0.657315 |
| S_31_ | 0.647265 | 0.810376 | 0.558035 | 0.636807 |
| S_41_ | 0.692117 | **0.880237** | **0.659241** | 0.697779 |
| S_02_ | **0.736387** | 0.722527 | 0.606531 | **0.733615** |
| S_12_ | 0.703984 | 0.767895 | **0.659241** | **0.712292** |
| S_22_ | 0.709638 | **0.830359** | **0.716531** | **0.735161** |
| S_32_ | **0.713195** | **0.875735** | **0.659241** | **0.729517** |

**Table S5** Band-integrated radiance of S32 and oxide substrates in 3-5 µm

| T /K  BIR  /W·m^-2^  Cases | 300 K | 600 K | 900 K | 1200 K | 1500 K |
| --- | --- | --- | --- | --- | --- |
|  | TPMs/Subs | TPMs/Subs | TPMs/Subs | TPMs/Subs | TPMs/Subs |
| MS-1km | 0.0037/0.013 | 1.35/5.53 | 8.92/44.43 | 24.81/129.82 | 54.43/253.32 |
| MS-8km | 0.29/0.68 | 79.45/259.26 | 504.51/2074.53 | 1375.58/6083.57 | 2923.89/11913.37 |
| MS-15km | 0.57/1.2 | 14.21/41.34 | 888.25/3282.59 | 2402.24/9624.43 | 5034.31/18864.51 |
| Tp-1km | 0.0023/0.010 | 1.02/4.65 | 6.98/37.51 | 19.42/109.65 | 43.31/213.94 |
| Tp-1km | 0.21/0.57 | 63.73/229.40 | 411.51/1844.07 | 1132.49/5407.40 | 2440.97/10582.25 |
| Tp-1km | 0.45/1.01 | 119.66/374.53 | 754.74/2980.03 | 2050.75/8726.08 | 4336.86/17080.21 |
| SW-1km | 0.034/0.049 | 6.71/13.71 | 39.84/104.59 | 104.43/303.84 | 208.78/594.17 |
| SW-8km | 0.83/1.29 | 174.33/411.56 | 1078.50/3290.34 | 2908.18/9765.39 | 5979.95/19322.46 |
| SW-15km | 1.20/1.87 | 253.00/589.33 | 1572.07/4721.80 | 4254.71/14050.00 | 8763.18/27854.49 |

**Table S6** Radiation suppression efficiency and radiation attenuation values of S32 and oxide substrates

| Cases | 300 K | | 600 K | | 900 K | | 1200 K | | 1500 K | |
| --- | --- | --- | --- | --- | --- | --- | --- | --- | --- | --- |
|  | η /% | I_dB_ /dB | η /% | I_dB_ /dB | η /% | I_dB_ /dB | η /% | I_dB_ /dB | η /% | I_dB_ /dB |
| MS-1km | 72% | -5.5 | 76% | -6.2 | 80% | -7.0 | **81%** | **-7.2** | **79%** | **-6.8** |
| MS-8km | 58% | -3.8 | 69% | -5.1 | 76% | -6.2 | **77%** | **-6.4** | **75%** | **-6.0** |
| MS-15km | 51% | -3.1 | 64% | -4.7 | 73% | -5.7 | **75%** | **-6.0** | **73%** | **-5.7** |
| Tp-1km | 78% | -6.6 | 78% | -6.6 | 81% | -7.2 | **82%** | **-7.4** | **80%** | **-7.0** |
| Tp-8km | 64% | -4.4 | 72% | -5.5 | 78% | -6.6 | **79%** | **-6.8** | **77%** | **-6.4** |
| Tp-15km | 56% | -3.6 | 68% | -4.9 | 75% | -6.0 | **77%** | **-6.4** | **77%** | **-6.0** |
| SW-1km | 30% | -1.5 | 51% | -3.1 | 62% | -4.2 | 66% | -4.7 | 65% | -4.6 |
| SW-8km | 36% | -1.9 | 58% | -3.8 | 67% | -4.8 | 70% | -5.2 | 69% | -5.1 |
| SW-15km | 36% | -1.9 | 57% | -3.7 | 67% | -4.8 | 70% | -5.2 | 69% | -5.1 |
